# Comparative transcriptome analysis uncovers roles of hydrogen sulfide for alleviating cadmium toxicity in *Tetrahymena thermophila*

**DOI:** 10.1186/s12864-020-07337-9

**Published:** 2021-01-06

**Authors:** Hongrui Lv, Jing Xu, Tao Bo, Wei Wang

**Affiliations:** 1 School of Life Science, Shanxi University, Taiyuan, 030006 China; 2 Key Laboratory of Chemical Biology and Molecular Engineering of Ministry of Education, Institute of Biotechnology, Shanxi University, Taiyuan, 030006 China

**Keywords:** *Tetrahymena thermophila*, Cd stress, H_2_S, Transcriptome, Oxidation resistance, Regulation of transport

## Abstract

**Background:**

Cadmium (Cd) is a nonessential heavy metal with potentially deleterious effects on different organisms. The organisms have evolved sophisticated defense system to alleviate heavy metal toxicity. Hydrogen sulfide (H_2_S) effectively alleviates heavy metal toxicity in plants and reduces oxidative stress in mammals. However, the function of H_2_S for alleviating heavy metal toxicity in aquatic organisms remains less clear. *Tetrahymena thermophila* is an important model organism to evaluate toxic contaminants in an aquatic environment. In this study, the molecular roles of exogenously H_2_S application were explored by RNA sequencing under Cd stress in *T. thermophila*.

**Results:**

The exposure of 30 μM Cd resulted in *T. thermophila* growth inhibition, cell nigrescence, and malondialdehyde (MDA) content considerably increase. However, exogenous NaHS (donor of H_2_S, 70 μM) significantly alleviated the Cd-induced toxicity by inhibiting Cd absorbtion, promoting CdS nanoparticles formation and improving antioxidant system. Comparative transcriptome analysis showed that the expression levels of 9152 genes changed under Cd stress (4658 upregulated and 4494 downregulated). However, only 1359 genes were differentially expressed with NaHS treatment under Cd stress (1087 upregulated and 272 downregulated). The functional categories of the differentially expressed genes (DEGs) by gene ontology (GO) revealed that the transcripts involved in the oxidation–reduction process, oxidoreductase activity, glutathione peroxidase activity, and cell redox homeostasis were the considerable enrichments between Cd stress and NaHS treatment under Cd stress. Kyoto Encyclopedia of Genes and Genomes (KEGG) indicated that the carbon metabolism, glutathione metabolism, metabolism of xenobiotics by cytochrome P450, and ABC transporters were significantly differentially expressed components between Cd stress and NaHS treatment under Cd stress in *T. thermophila*. The relative expression levels of six DEGs were further confirmed through quantitative real-time polymerase chain reaction (qRT-PCR).

**Conclusion:**

NaHS alleviated Cd stress mainly through inhibiting Cd absorbtion, promoting CdS nanoparticles formation, increasing oxidation resistance, and regulation of transport in free-living unicellular *T. thermophila*. These findings will expand our understanding for H_2_S functions in the freshwater protozoa.

**Supplementary Information:**

The online version contains supplementary material available at 10.1186/s12864-020-07337-9.

## Background

Heavy metal contamination in aquatic environments has become a global issue [1]. Heavy metals cause adverse effects on the environment because of their toxicity, persistency, and nonbiodegradability [2]. Cadmium (Cd) is one of the most deleterious and serious environmental pollutant to animals and plants [3]. It directly disturbs protein structures and inhibits enzyme activities and causes the formation of reactive oxygen species (ROS), such as superoxide anion (O^•^_2_^−^), hydroxyl radical (OH^•^), and hydrogen peroxide (H_2_O_2_), which in turn induce oxidative stress and membrane damage [4]. ROS leads to serious damages to different macromolecules, such as DNA, RNA, proteins, and lipids [5]. To survive against the stresses, different organisms have evolved a complex of mechanisms involving multiple genes and strategies at physiological, molecular and metabolic levels, such as activating antioxidants, increasing efflux, and overexpressing metal chelators. The organisms effectively respond to ROS through enzymatic and nonenzymatic antioxidant systems [6]. Superoxide dismutase (SOD) is responsible for the conversion of superoxide radicals to H_2_O_2_. Catalase (CAT) decomposes H_2_O_2_ into H_2_O and O_2_ [7]. Glutathione (GSH) directly or indirectly protects against ROS-mediated cell injury. Several GSH-associated enzymes, such as glutathione reductase, glutathione peroxidase (GPX), and glutathione S-transferase (GST), cumulatively protect against ROS under toxic metal stress [8]. Recent studies revealed that exogenous gaseous signal molecule hydrogen sulfide (H_2_S) improve Cd tolerance in plants by reducing oxidative damage.

H_2_S is produced endogenously from cysteine mainly by cystathionine β-synthase (CBS) and cystathionine γ-lyase (CGL) and is important for various physiological functions in mammals, including synaptic transmission, vascular tone, inflammation, angiogenesis, and protection from oxidative stress [9]. Exogenous H_2_S acts as a potent antioxidant under Cd stress by enhancing antioxidant enzymes activities in wheat seedlings [10], and alleviates Cd toxicity through regulations of Cd transport across the plasma and vacuolar membranes in *Populus euphratica* cells [11]. In *Brassica rapa*, H_2_S mitigates Cd-induced cell death by inhibiting ROS accumulation [12]. H_2_S reduced Cd-induced oxidative stress, particularly by enhancing redox status and the activities of ROS and methylglyoxal detoxifying enzymes in rice [13]. However, the functions and signal pathways of H_2_S under heavy metal stress remain unclear in other organisms.

Ciliates are highly divergent unicellular eukaryotic organisms with nuclear dualism. These unicellular eukaryotic organisms are ubiquitous in various environments [14]. Ciliates play an important role in aquatic ecosystem and are used as whole cell biosensors to evaluate toxicity of various environmental pollutants [15, 16]. *Tetrahymena thermophila* is a free-living ciliate widely distributed in freshwater environment. It is an excellent model organism for toxicological and ecotoxicological studies in aquatic toxicity test systems. *T. thermophila* contains a large number of gene families that are involved in processes associated with sensing and responding to environmental stresses. The 44 P450 monooxygenase genes and 165 ATP-binding cassette (ABC) transporter genes were identified in *T. thermophila* [17, 18]. The 70 putative GST genes exist in the macronuclear genome of *T. thermophila*, which imply that this organism has been exposed to diverse xenobiotics throughout its evolution [19]. *T. thermophila* has higher sensitivity to heavy metal stress [20]. One of the basic heavy metal resistance mechanisms present in *T. thermophila* is the intracellular sequestration (bio-accumulation), of which the cellular detoxification processes is the chelation of metal cations by endogenous proteins or peptides, such as metallothioneins (MTs), phytochelatins (PCs), and GSH [14]. Heavy metal stress responsive genes and antioxidant defense system allow the survival of *Tetmemena* in presence of metals in the environment [21]. These heavy metal stress response mechanisms in freshwater ciliates are kinds of self-protection through intracellular regulation.

However, little is known about the mitigative effects on heavy metal stress through exogenous additive in the freshwater protozoa. In this study, we found H_2_S promoted *T. thermophila* proliferation and alleviated cellular toxicity induced by Cd. The mechanism of H_2_S function on *T. thermophila* stressed under Cd was evaluated by phenotypic observation, enzyme and metabolites analysis, and high throughput transcriptome sequencing technology. These findings will expand our understanding for H_2_S functions in aquatic organisms.

## Results

### H_2_S mitigates inhibition of proliferation of *T. thermophila* under Cd stress

**Fig. 1:**
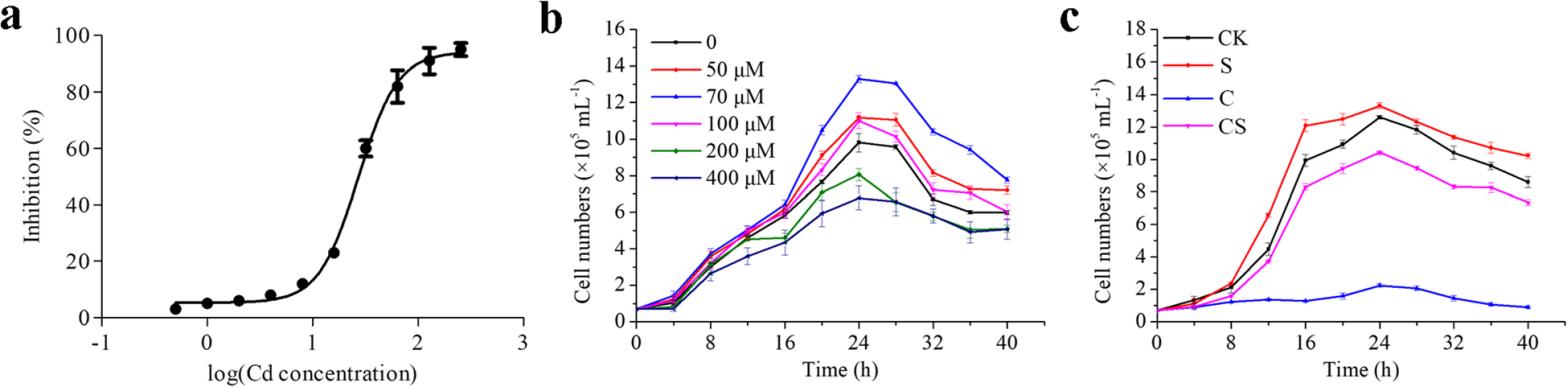
Proliferation of *T. thermophila* under Cd and H_2_S treatments. **a** IC50 value of Cd. The inhibition ratio of cell proliferation in different concentrations of Cd (0.5, 1, 2, 4, 8, 16, 32, 64, 128, and 256 μM). The IC50 of Cd was calculated by GraphPad Prism 5. **b** Cell proliferation in different concentrations of NaHS (50, 70, 100, 200, and 400 μM). **c** Cell proliferation under 70 μM NaHS (S), 30 μM Cd (C), and 30 μM Cd+ 70 μM NaHS (CS) treatments

Heavy metal pollutants caused toxic effects on ciliates, and the effect varied according to the bioavailable concentration and nature of the heavy metal [22]. An assay using the motile response of *Tetrahymena pyriformis*, gave a sensitivity better than 1 μM and a toxicity threshold to 7 μM for Cd [23]. Cd caused a dose-dependent decline in the viability of *T. thermophila* [24]. To understand the tolerance level of Cd for *T. thermophila*, the half maximal inhibitory concentration (IC50) value of Cd was determined, and it was calculated to be 30 μM for *T. thermophila* cells at 6 h culture (Fig. 1a)*.* H_2_S alleviates Cd toxicity in plants. Exogenous H_2_S recovered Cd-induced growth inhibition in *Brassica napus*, *Arabidopsis*, and barley [25–27]. 70 μM NaHS (donor of H_2_S) largely stimulated proliferation of *T. thermophila* (Fig. 1b). Furthermore, the proliferation of *T. thermophila* under Cd and NaHS treatments was investigated. An amount of 0.7× 10^5^ mL^− 1^ cells was transferred to the SPP medium, and the number of cells was counted every 4 h. The proliferation inhibition of *T. thermophila* under 30 μM Cd was dramatically mitigated by 70 μM NaHS (Fig. 1c). The results showed exogenous NaHS play a protective role on Cd stress in *T. thermophila*.

### Cd treatment promotes the production of endogenous H_2_S and cysteine in *T. thermophila*

**Fig. 2:**
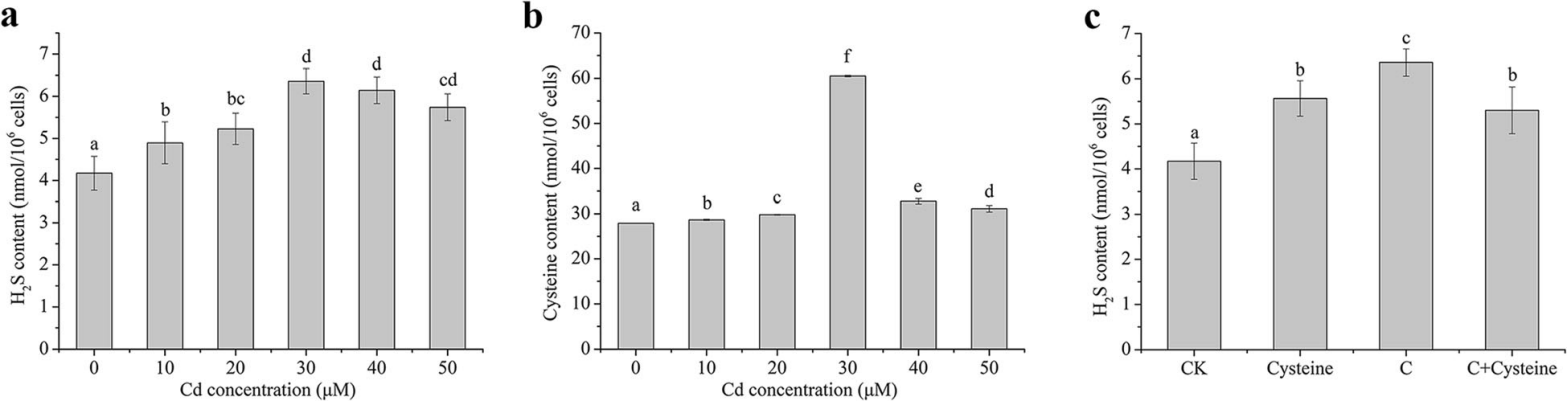
Analysis of endogenous H_2_S and cysteine contents in *T thermophila*. **a** Changes of H_2_S content in various Cd concentrations. **b** Changes of cysteine content in various Cd concentrations. **c** The effects of cysteine on H_2_S content under Cd stress. Cells were treated with 1 mM cysteine, 30 μM Cd (C), and 30 μM Cd+ 1 mM cysteine (C+Cysteine) for 6 h. Data are means ± SE of three biological repeats, error bars indicate error standard. Means denoted by the same letter were not significantly different at *P* > 0.05, and different letters indicate statistically significantly differences (*P* < 0.05) by Duncan Multiple Range Test (DMRT)

Endogenous H_2_S is generated through enzymatic pathways in plants. Cysteine desulfhydrases regulate cysteine degradation into pyruvate, ammonia and H_2_S. In contrast, O-acetylserine (thiol) lyase catalyzes the formation of cysteine using H_2_S and O-acetylserine. These physiological processes are interrelated under Cd stress [26]. Recently, we also found cysteine is generated by reverse transsulfuration pathway involved CBS and CGL, and de novo pathway involved cysteine synthase (CS) in *T. thermophila*. At the same time, the CBS, CGL, and CS also catalyzed H_2_S production in vitro [28]. To explore whether endogenous H_2_S is involved in *T. thermophila* tolerance to Cd stress, formation of endogenous H_2_S was investigated under different conditions. 10 to 30 μM Cd increased the H_2_S content and cysteine levels in a dose-dependent manner. When *T. thermophila* cells were treated with 40 or 50 μM Cd, both H_2_S and cysteine levels decreased due to stronger Cd toxicity (Fig. 2a, b). Exogenous cysteine treatment enhanced H_2_S level and maintained H_2_S at high level under Cd stress (Fig. 2c).

### H_2_S alleviates lipid peroxidation and improves antioxidant capacity under Cd stress

**Fig. 3:**
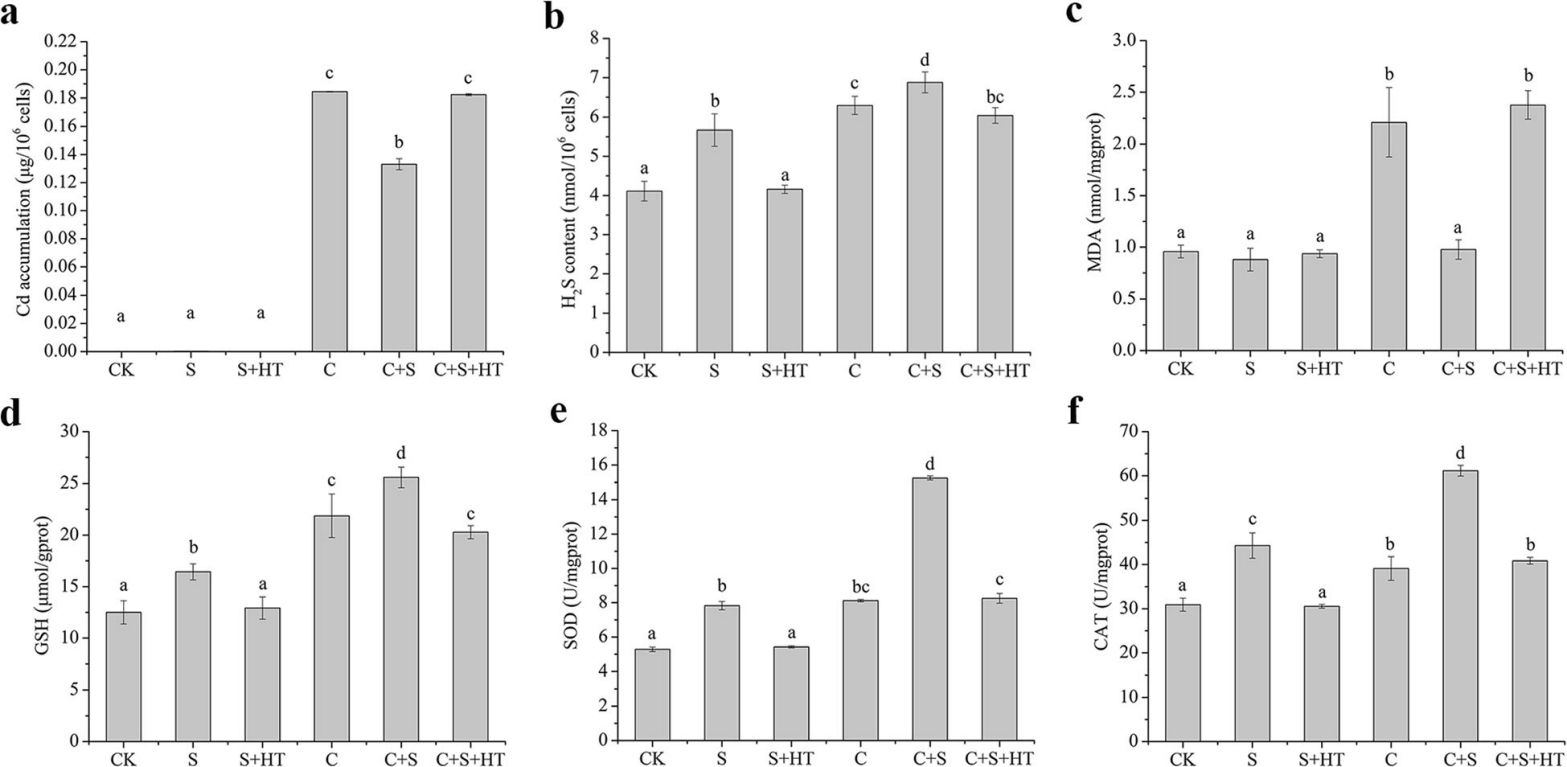
Effects of NaHS on Cd accumulation, H_2_S content, lipid peroxidation, and antioxidant system under Cd stress. **a** Cd accumulation under different conditions in *T. thermophila*. **b** H_2_S contents in *T. thermophila* cells when the cells were treated by various conditions. **c** Effects of Cd and H_2_S on lipid peroxidation. **d**-**f** Cells in the logarithmic phase were grown in the medium with different treatments, and GSH content (**d**), SOD activity (**e**), and CAT activity (**f**) were measured. CK, S, S+HT, C, C+S, and C+S+HT correspond to the groups of cells exposed to nutrients only, at 70 μM NaHS, 70 μM NaHS+ 140 μM HT, 30 μM CdCl_2_, 30 μM CdCl_2_+ 70 μM NaHS, and 30 μM CdCl_2_+ 70 μM NaHS + 140 μM HT respectively. Data are means ± SE of three biological repeats, error bars indicate error standard. Means denoted by the same letter were not significantly different at *P* > 0.05, and different letters indicate statistically significantly differences (*P* < 0.05) by Duncan Multiple Range Test (DMRT)

Cd significantly inhibits the growth of microorganisms and plants. The treatment using 30 μM Cd also led to the stunted growth and nigrescence of *T. thermophila* after being exposed for 24 h. However, the toxic symptoms were drastically alleviated with NaHS supplement. The exogenous NaHS significantly inhibited Cd accumulation (decrease by 26%) in the *T. thermophila* cells (Fig. 3a). The H_2_S-mediated Cd accumulation was significantly decreased with hypotaurine (HT, a H_2_S scavenger that reverses the effect of H_2_S) treatment. NaHS application increased H_2_S level by 38% but did not affect it in H_2_S + HT group compared to untreated control (Fig. 3b). The results implied the regulatory role of H_2_S in Cd accumulation in *T. thermophila*.

Cd stress caused lipid peroxidation and induced malondialdehyde (MDA) production of *T. thermophila* cells in a dose-dependent manner. Low concentrations of Cd had less effect on the MDA content in *T. thermophila* cells. However, 30 and 50 μM Cd lead to the MDA content of the cells increased by 91 and 395%, respectively (Fig. S1). In comparison, the MDA content of the cells had no significant changes when the cells were treated with NaHS. However, the NaHS treatment markedly decreased the MDA content of *T. thermophila* cells under Cd stress (Fig. 3c).

It is well known that antioxidant defense system increases organism tolerance against metal-induced toxicity by upregulating the nonenzymatic antioxidants and different antioxidant enzymes. H_2_S increase GSH content and antioxidant enzymes activity in *Arabidopsis* [26]. Under Cd stress, GSH content increased by 51% and exogenous NaHS supplement further increased the GSH content in *T. thermophila* (Fig. 3d). But, the increase of GSH was reversed with HT supply. Furthermore, SOD activity increased by 92% and CAT activity increased by 29% under Cd stress. H_2_S supplement also enhanced SOD and CAT activities in *T. thermophila* cells (Fig. 3e, f). Combined application of NaHS and HT decreased the activities of SOD and CAT. The results indicated that H_2_S could alleviate Cd toxicity by improving the antioxidant capacity of *T. thermophila* cells.

**Fig. 4:**
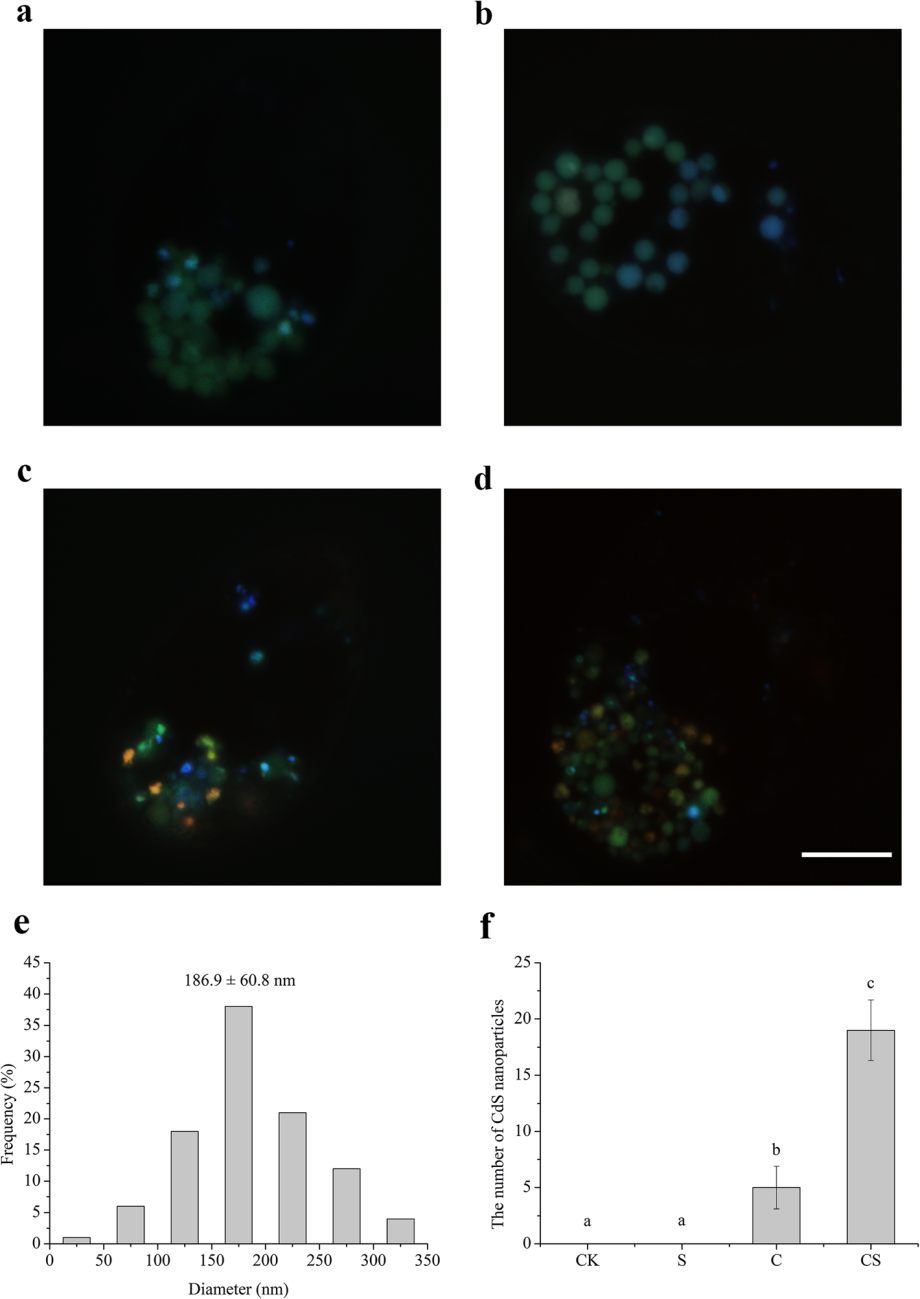
Images of live *T. thermophila* cells under ultraviolet excitation light. Cells were exposed to nutrients only (**a**), 70 μM NaHS (**b**), 30 μM Cd (**c**), and 30 μM Cd+ 70 μM NaHS (**d**). Scale bar = 10 μm. **e** histogram of particle size distribution obtained from corresponding images. **f** The number of CdS nanoparticles with different treatments (*n* = 25)

NaHS increased the insoluble Cd fractions in salix leaves and roots [29]. *Schizosaccharomyces pombe* directly scavenge the free Cd^2+^ ions and the detoxification process occurs through the production of CdS nanoparticles [30]. In *T. thermophila,* spherical CdS nanoparticles in yellow colour with an average particle diameter of 186.9 ± 60.8 nm were observed under Cd treatment, and the nanoparticles amount increased by adding NaHS (Fig. 4). However, UV-visible spectrum analysis showed that no CdS formation was found in vitro (Fig. S2). The formation of Ag nanoparticles from Ag ions was one of the defense mechanisms of *T. thermophila* against the toxic silver ions. Compared to AgNO_3_, Ag nanoparticles were remarkably less toxic. The Ag nanoparticles stored intracellularly in the food vacuoles of *T. thermophila* [31]. The results showed that the formation of CdS nanoparticles decrease Cd bioavailability and toxicity in *T. thermophila*.

### Characterization of the sequenced Illumina libraries

*Tetrahymena* evolved various efficient detoxification pathways allowing the survival from heavy metal stress, such as overexpressing metal chelators and activating antioxidant signal pathways. The oxidative stress related mechanism of Ag nanoparticles was revealed at the transcriptional level. Some oxidative stress related genes were upregulated upon exposure to sub-lethal concentrations of Ag compounds, although intracellular ROS levels and SOD and CAT activities were not elevated in *Tetrahymena* [32]. To further explore the mechanisms of H_2_S alleviating Cd stress in *T. thermophila*, RNA-seq was employed to investigate the changes in genome-wide gene expression for four groups of cells: exposed to nutrients only (CK), with 70 μM NaHS (S), under 30 μM CdCl_2_ (C), and 70 μM NaHS+ 30 μM CdCl_2_ (CS), with three biological replicates. A total of 305.2 million pair-end reads with Q20 > 97% and Q30 > 93% were obtained from 12 libraries (Table 1). Among the short clean reads, more than 94% were mapped to the *T. thermophila* Functional Genomics Database (http://tfgd.ihb.ac.cn/). Approximately 95% of the reads from each library were perfectly matched to the reference genes, and more than 93% of the reads in the libraries were mapped to single locations. Half of these uniquely mapped reads in each library were mapped to the sense strand, whereas the other half was mapped to the antisense strand (Table S1). Then, the mapped reads were further classified and annotated using TopHat [33]. The correlations between the three replicated samples were calculated on the basis of the normalized expression results (Fig. S3). The correlation coefficient between the three replicated samples was reasonable for the CK, S, and CS groups, but that between C1 and C2 or between C1 and C3 was lower than 70%. Thus, the C1 sample was abnegated.

**Table 1:** Summary statistics of transcriptome sequencing

Sample name	Pair-end reads	Base sum	Q20 (%)	Q30 (%)	GC content (%)
CK1	24,039,989	7.18 G	97.62	93.23	34.4
CK2	26,249,793	7.85 G	97.45	92.89	35.38
CK3	27,398,030	8.18 G	97.76	93.58	36.48
S1	24,065,268	7.20 G	97.78	93.65	36.46
S2	25,128,738	7.51 G	97.67	93.36	34.23
S3	27,739,667	8.26 G	97.77	93.57	34.89
C1	24,615,672	7.36 G	97.68	93.35	33.98
C2	24,782,234	7.42 G	97.64	93.2	33.22
C3	24,731,403	7.39 G	97.68	93.32	33.39
CS1	22,918,319	6.86 G	97.72	93.44	34.4
CS2	26,462,241	7.90 G	97.75	93.55	35.38
CS3	27,065,424	8.08 G	97.7	93.39	36.48

### Differentially expressed genes (DEGs) in response to NaHS treatment, Cd stress, and NaHS treatment under Cd stress

**Fig. 5:**
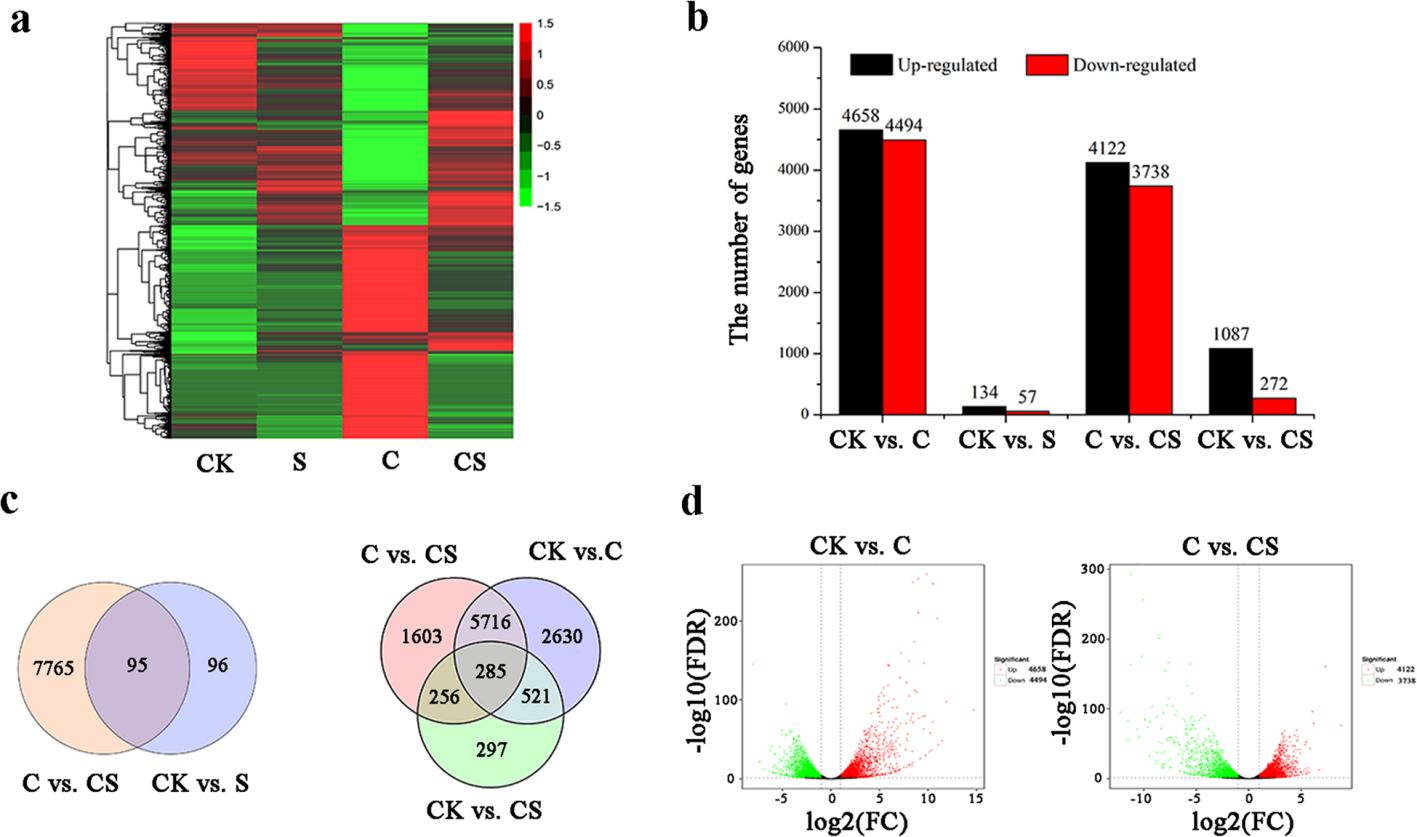
Responses of DEGs to H_2_S treatment, Cd stress, and H_2_S treatment under Cd stress. **a** Hierarchical clustering of all DEGs based on log10 fragments per kilobase million (FPKM) values. The color from green to red represents the gene expression level from low to high. **b** Distribution of upregulated and downregulated transcripts in each comparison. **c** Venn diagram analysis of differentially expressed transcripts between four pairwise comparisons. ) Volcano plots of DEGs between samples. The threshold q < 0.05 was used to determine the significance of DEGs. Red and green dots represent up- and down-regulated genes, respectively, and black dots indicate transcripts that did not change significantly in the CK vs. C or C vs. CS

DEGs were hierarchically clustered to obtain a comprehensive view of the differential gene expression under NaHS treatment, Cd stress, and NaHS treatment with Cd stress (Fig. 5a). Under NaHS treatment, the expression level of 191 genes changed. Among them, 134 genes were upregulated and 57 genes were downregulated. Under Cd stress, the expression level of 9152 genes significantly changed, including 4658 upregulated genes and 4494 downregulated genes. A total of 1087 genes were upregulated and 272 genes were downregulated with NaHS treatment under Cd stress. The expression levels of most genes recovered under Cd stress with NaHS treatment. A total of 4122 genes were upregulated and 3738 genes were downregulated between Cd stress and NaHS treatment under Cd stress (Fig. 5b).

The 95 DEGs overlapped between CK vs. S (50.0%) and C vs. CS (1.2%), indicating that H_2_S-responsive transcripts under normal condition were far fewer than those under Cd stress. The 806 DEGs in CK vs. C (8.8%) were common to CK vs. CS (59.3%), and the 6001 genes overlapped between CK vs. C (65.6%) and C vs. CS (76.3%) (Fig. 5c), suggesting most of the Cd-responsive transcripts were altered by H_2_S. The absolute value of the log2 ratio ranged from 1.00 to 14.70 in CK vs. C, and ranged from 1.00 to 12.21 in C vs. CS (Fig. 5d). The significantly upregulated genes under Cd stress (log2FC > 8) were considerably related to oxidoreductase, GPXs, GSTs, heat shock protein, and MTs (Table S2). By systematic bioinformatics approach, the predicted *T. thermophila* Cd proteome included thioredoxins, heat shock proteins, GPXs, GSTs, and MT protein [34]. Compared with Cd stress, the unigenes significantly downregulated in the NaHS treatment under Cd stress (log2FC < − 8) were mainly related to oxidoreductase, GPXs, GSTs, and heat shock protein (Table S3). The results indicated that the redox system is sensitive for NaHS treatment and Cd stress in *T. thermophila*.

### Gene ontology (GO) enrichment analysis of DEGs

To obtain the functional annotations of the DEGs for Cd stress and H_2_S treatment under Cd stress, GO category enrichment analysis was performed. For the comparison of CK vs. C, the 5740 DEGs were classified as 50 functional groups (Fig. S4). The functional groups were divided into three categories: biological process, molecular function, and cellular component. The biological process mainly comprises DEGs involved in metabolic process (2619, 45.63%), cellular process (2589, 45.10%), single-organism process (1406, 24.49%), biological regulation (777, 13.5%), and localization (706, 12.30%). In the category of cellular components, membrane (2711, 47.23%), membrane part (2517, 43.85%), cell (1798, 31.32%), cell part (1784, 31.08%), and organelle (1094, 19.06%) were the most represented groups. Among the molecular function category, the major groups were catalytic activity (3081, 53.68%), binding (2160, 37.63%), and transporter activity (416, 7.25%). For the comparison of C vs. CS, the 4883 DEGs were also classified as 50 functional groups. Between C vs. CS and CK vs. C, they had exactly identical classification patterns (Fig. S4).

**Fig. 6:**
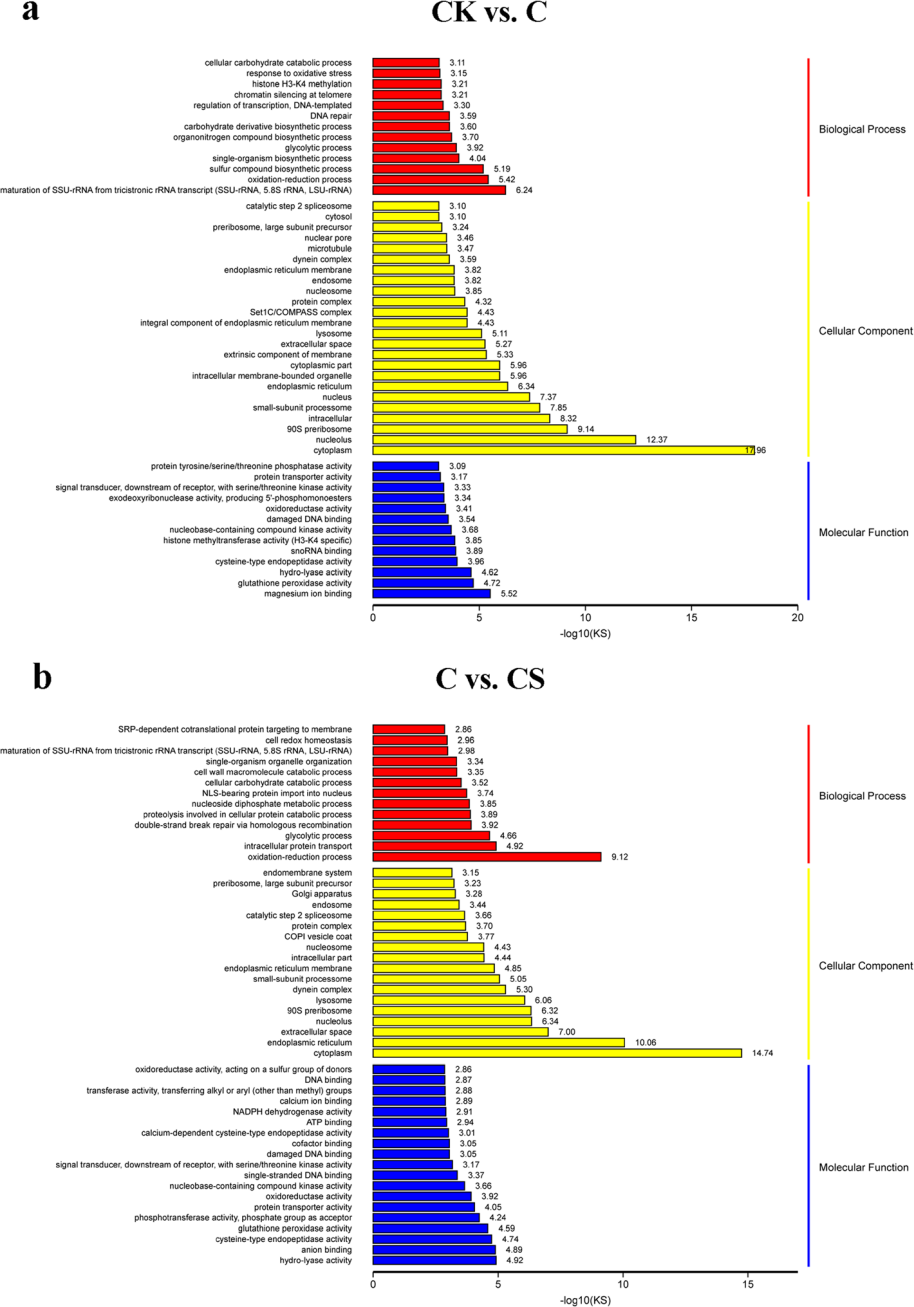
TopGO enrichment analysis of DEGs. **a** TopGO enrichment analysis for GO in CK vs. C. **b** TopGO enrichment analysis for GO in C vs. CS. The ordinate coordinates represent three GO categories under the GO term level, including the annotation of the term and the abscissa is the enriched degree. The low KS result indicates more significant enrichment

Next, TopGO enrichment analysis was performed to obtain a detailed classification through false discovery rate (FDR) adjusted *P*-value of < 0.05 as the cutoff (Fig. 6). The distribution of enriched GO terms indicated that several DEGs were involved in oxidation–reduction process (GO:0055114), oxidoreductase activity (GO:0016491) and glutathione peroxidase activity (GO:0004602) in both CK vs. C and C vs. CS. Under Cd stress, 283 DEGs were included in oxidoreductase activity and 231 DEGs participated in oxidation-reduction process. Compared with Cd stress, 263 DEGs constituted the oxidoreductase activity with NaHS addition, and 182 DEGs involved in oxidation–reduction process. Furthermore, response to oxidative stress (GO:0006979) was enriched in CK vs. C. Cell redox homeostasis (GO:0045454) was enriched in C vs. CS. These data indicated that H_2_S responds to Cd stress mainly through the adjustment of the redox balance.

### Kyoto encyclopedia of genes and genomes (KEGG) metabolic pathway enrichment analysis

**Fig. 7:**
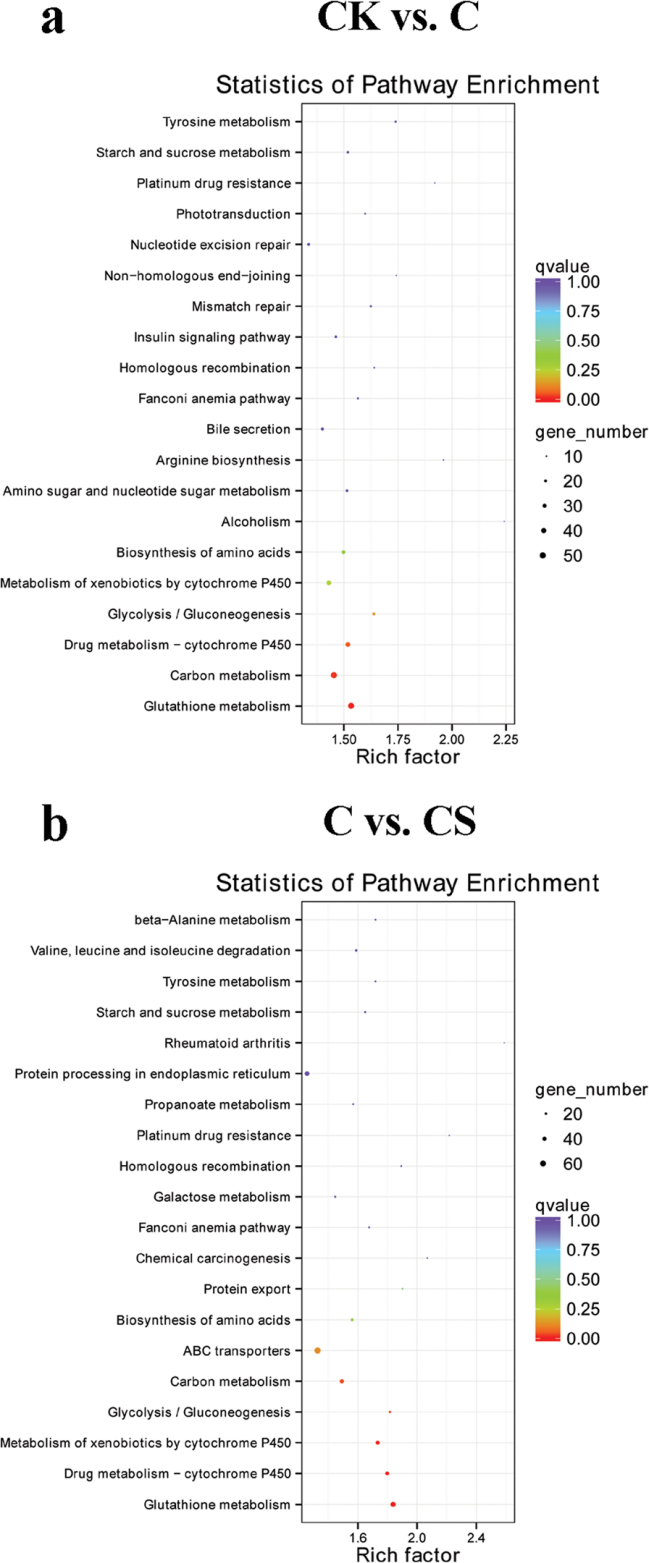
KEGG pathway enrichment analyses of DEGs in CK vs. C (**a**) and C vs. CS (**b**). The left Y-axis shows the KEGG pathway. The X-axis shows the enrichment factor. The enrichment factor indicates the ratio of differentially expressed unigenes enriched in this pathway to the total number of annotated unigenes. The size and color of each point represent the number of genes enriched in a particular pathway. A large enrichment factor value and low q-values indicate a large degree of enrichment

The annotated *T. thermophila* transcripts were mapped to the KEGG pathways to investigate the genes involved in important metabolic pathways. Under Cd stress, the 1116 DEGs were mapped to the 252 KEGG pathways. For NaHS treatment under Cd stress compared with Cd stress, 966 DEGs were mapped to 247 KEGG pathways. The pathways considerably related to carbon metabolism, GSH metabolism, drug metabolism–cytochrome P450, and metabolism of xenobiotics by cytochrome P450 (Fig. 7a, b). Under Cd stress, 54 DEGs (4.84%) were distributed in the carbon metabolism, and 48 DEGs (4.97%) also enriched in this pathway with adding NaHS. The KEGG pathway of GSH metabolism includes primarily GPX and GST. 52 DEGs (4.66%) were enriched at the GSH metabolism under Cd stress, and 54 DEGs (5.59%) were also enriched with adding NaHS. Under Cd stress, 39 DEGs (3.49%) or 40 DEGs (3.58%) were distributed in drug metabolism–cytochrome P450 or metabolism of xenobiotics by cytochrome P450, and 40 DEGs (4.14%) or 42 DEGs (4.35%) were enriched in the two pathways with NaHS addition, respectively. Besides, under Cd stress, 67 DEGs (6.00%) were distributed in ABC transporters (Fig. S5), and 70 DEGs (7.25%) were enriched in this pathway with adding NaHS. The considerably enriched pathways in C vs. CS were similar to those of CK vs. C, indicating the important detox effects through H_2_S under Cd stress.

### Regulation of cytochrome P450 and GSH metabolism under Cd stress and with NaHS treatment

Cytochrome P450 represents an important participant in regulatory networks of organism responses to Cd stress. The expression of genes encoding cytochrome P450 family proteins was strongly induced by Cd in rice [35]. In the wolf spider *Pardosa pseudoannulata*, cytochrome P450 genes were found to respond to Cd stress [36]. A total of 44 putative functional cytochrome P450 genes were identified and classified into 13 families and 21 sub-families according to standard nomenclature in *T. thermophila*. Cd induced the expression of 6 cytochrome P450 genes and inhibited the expression of 9 cytochrome P450 genes (|Log2FC|> 1). NaHS addition reversed the expression of 11 of them (Table 2). Under Cd stress, metabolism of xenobiotics by cytochrome P450 was significantly enriched by KEGG pathway analysis on DEGs, of which 32 DEGs were markedly upregulated (Fig. S6), including oxidoreductase, aldehyde dehydrogenase, 30 GSTs, and 29 of them were markedly downregulated when NaHS was added.

**Table 2:** Cytochrome P450 genes response to Cd or H_2_S in CK vs. C and/or C vs. CS

Gene ID	Gene name	CK vs. C Log2FC	C vs. CS Log2FC
TTHERM_00191380	CYP5004A1	−1.222948644	0.726722049
TTHERM_00198210	CYP5005A2	−2.013242365	1.999422405
TTHERM_00198230	CYP5005A4	−1.097798649	0.315367456
TTHERM_00201580	CYP5005A9	3.180647522	−3.21680049
TTHERM_00201630	CYP5005A10	2.330671223	−1.440492726
TTHERM_00898330	CYP5005A15	2.830883564	−2.410899716
TTHERM_01122770	CYP5005A18	−1.082132664	0.922173282
TTHERM_01122780	CYP5005A19	−1.307720517	1.50630203
TTHERM_00185610	CYP5006A1	−2.139546793	1.632428135
TTHERM_00283410	CYP5007B1	1.820581108	−1.11407977
TTHERM_00444460	CYP5009A1	2.445007531	−1.43647057
TTHERM_00723150	CYP5010A1	−1.023465363	0.416122088
TTHERM_00527100	CYP5011A1	−1.608260175	2.51348553
TTHERM_00241770	CYP5013C2	−2.841933829	2.710002866
TTHERM_00313500	CYP5013E1	1.277727898	−1.197335044

Sixty-three cytosolic GSTs have been identified in *T. thermophila*. Under Cd stress, GSH metabolism was significantly enriched by KEGG pathway analysis on upregulated genes (Fig. S7). The upregulated DEGs included 31 GSTs and 8 GPXs. Compared with Cd stress, GSH metabolism was also significantly enriched on downregulated genes with NaHS addition, including 30 GSTs and 8 GPXs. Specifically, the 8 upregulated GPXs and 29 GSTs induced by Cd stress returned to normal level with adding NaHS. GSTs detoxify xenobiotic compounds by linking the -SH group of antioxidant GSH covalently to a substrate [37]. Given the role of GSH in the detoxification of heavy metals, regulation of GSH, GPXs, and GSTs by H_2_S play an important role in ameliorating Cd stress.

### Regulation of ABC transporters under Cd stress and with NaHS treatment

GSH marked xenobiotic molecules are excreted from cells via phase III proteins such as ABC transporter enzymes. Based on the KEGG pathway analysis, the ABC transporters related to detoxification of Cd in *T. thermophila* was screened and listed in Fig. S8. Under Cd stress, 49 DEGs involved in the enrichment of ABC transporters were markedly upregulated, while 18 DEGs were downregulated. The ABC transporter family of *T. thermophila* was classified into 8 distinct groups [18]. The ABCA subfamily comprises of a total of 32 members, which included 9 upregulated genes and 3 downregulated genes under Cd stress. The ABCB subfamily consists of 26 transporters, of which 5 genes were upregulated and 5 genes were downregulated. The ABCC subfamily comprises 60 transporters and represents the largest family of ABC transporters in *T. thermophila*. Twenty-two genes were induced by Cd and 6 genes were downregulated. The ABCG subfamily includes 39 transporters, and 9 genes were upregulated under Cd stress and 3 genes were inhibited. ABCG19 (TTHERM_00034920) was induced up to 357 folds under Cd stress (Table S2), which suggested it could play an important role in detoxifying Cd toxicity. H_2_S has been demonstrated for direct regulating ABC transporters to induce stomatal movement in plants. Exogenous H_2_S induces stomatal closure and this effect is impaired by the ABC transporter inhibitor glibenclamide in guard cells [38]. Compared with Cd stress, 42 DEGs were downregulated and 28 DEGs were upregulated for ABC transporters with NaHS addition. Most of the expression of ABC transporters under Cd stress was restored and some of them even hyper activated by exogenous NaHS addition, which is in relation to H_2_S lowering the cellular concentration of Cd (Fig. 3a) and promoting CdS nanoparticles formation (Fig. 4).

### Quantitative real-time polymerase chain reaction (qRT-PCR) validation of differentially expressed transcripts

**Fig. 8:**
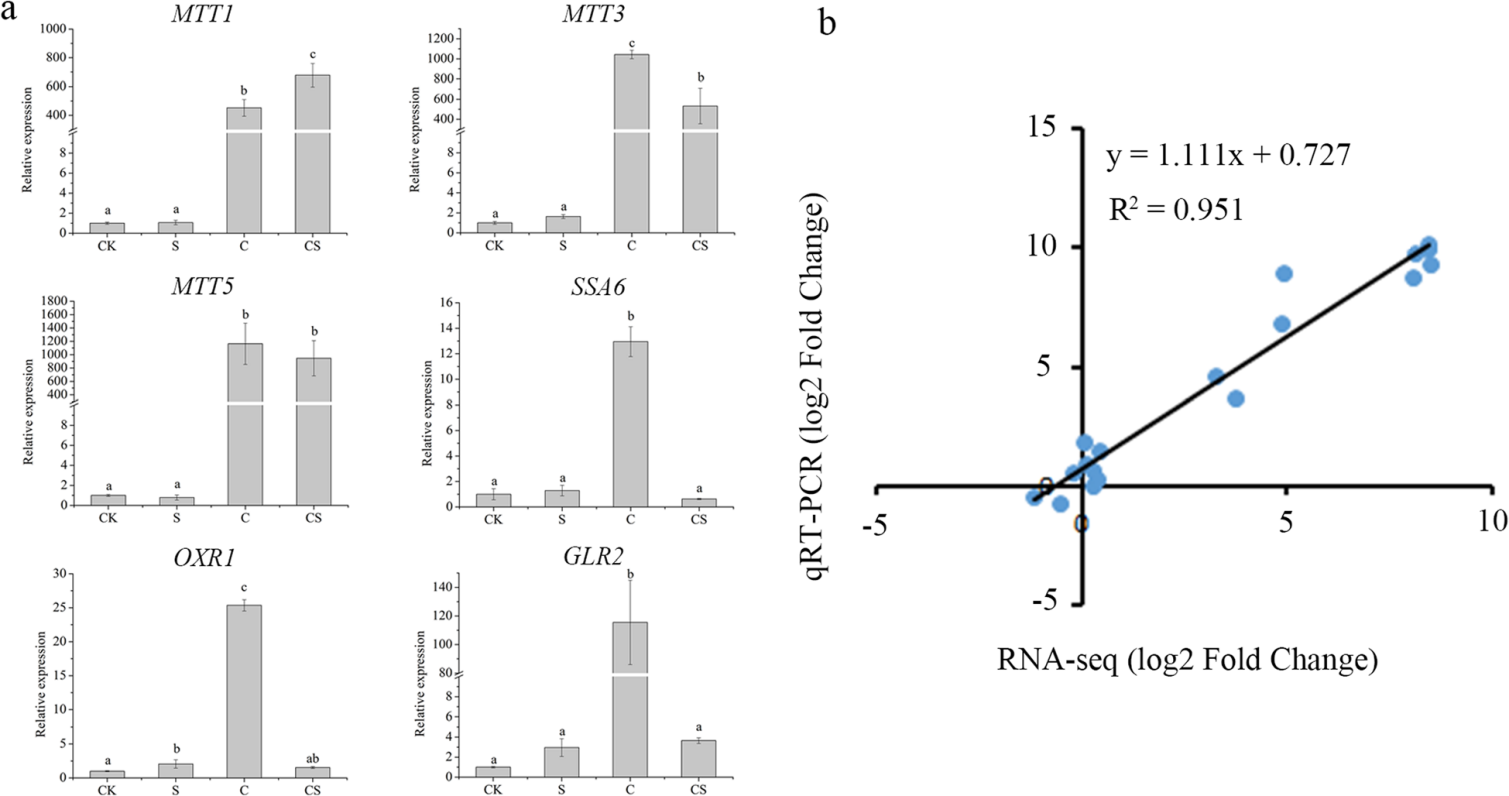
Relative expression levels of six DEGs with NaHS treatment and under Cd stress. **a** The six genes expression as determined by qRT-PCR. *MTT1* (TTHERM_00241640), *MTT3* (TTHERM_00241650), *MTT5* (TTHERM_00660230), oxidoreductase *OXR1* (TTHERM_00151670), thioredoxin and glutathione reductase *GLR2* (TTHERM_00047660), and dnak protein *SSA6* (TTHERM_00171850). **b** Comparison between the log2 of gene expression ratios obtained from RNA-seq data and qRT-PCR. The qPCR log2 value of the expression ratio (S/CK, C/CK, CS/CK) (y-axis) was plotted against the value from the RNA-seq (x-axis). R^2^ represented the correlation coefficient between qRT-PCR and RNA-seq

To validate the accuracy and reliability of transcriptome sequencing, six DEGs involved in chelating heavy metals, redox reaction, and stress response were evaluated through qRT-PCR. NaHS slightly affected the expression of the genes under non-stressed condition. Under Cd stress, the expression levels of the genes were significantly upregulated. The log2FC of the CdMT genes were ranked as *MTT5*≈*MTT3*>*MTT1* under Cd stress (Table S2). The expression levels of four genes decreased with NaHS addition, including *SSA6*, *OXR1*, *GLR2*, and *MTT3*. On the contrary, the expression level of *MTT1* further increased and *MTT5* had no obvious change with NaHS treatment under Cd stress (Fig. 8). The three CdMT isoform genes present differential expression patterns between Cd stress and NaHS treatment under Cd stress, indicating their functional diversification. The correlation between qRT-PCR and RNA-seq was measured by scatter plotting log2-fold changes, which showed a positive correlation coefficient in both techniques (Pearson coefficient R^2^ = 0.95), thereby indicating the reliability of the sequencing data.

## Discussion

### NaHS treatment improved Cd tolerance in *T. thermophila*

Cd is a nonessential and highly toxic metal that adversely affects the growth and development of different living organisms. 50–150 μM Cd inhibited the elongation of *Arabidopsis* roots in a dose-dependent manner [26]. Survival rate of three ciliates decreased remarkably with increasing Cd exposure, and the LC50 for *Notohymena* (5 μg/mL) was higher than *Tetmemena* (2 μg/mL) and *Euplotes* (2 μg/mL) [39]. The LC50 of Cd for *T. thermophila* SB1969 strain was 44.5 μM for 24 h exposure [40]. In the present study, IC50 of Cd for *T. thermophila* was 30 μM for 6 h treatment. Several strategies have been developed to alleviate the toxicity of Cd. Among them, H_2_S participates in suppressing Cd stress. 100 μM H_2_S supplementation exhibited an inhibitory effect on Cd uptake and accumulation in rice [13]. 300 μM H_2_S alleviates Cd toxicity by changing Cd chemical forms and increasing the activities of antioxidant enzymes in salix [29]. We found that 70 μM NaHS promoted the proliferation of *T. thermophila* and alleviated Cd toxicity by inhibiting the accumulation of Cd in cells (Fig. 1, 3a). Furthermore, we also found that copper (Cu) inhibited the proliferation of *T. thermophila*, and NaHS alleviated its inhibitory effect (Fig. S9).

### H_2_S participates in oxidative damage scavenging systems in *T. thermophila*

The antioxidant systems of enzymatic and nonenzymatic antioxidants could immediately abolish ROS. H_2_S alleviates oxidative damage against copper stress in wheat, and toxic effects of lead on cotton antioxidant activity [41, 42]. In ROS scavenging pathways, O_2_^−^ is converted to H_2_O_2_ by SOD, and H_2_O_2_ is subsequently reduced to H_2_O and O_2_ by CAT. 30 μM Cd induced a sharp generation of MDA and enhanced activity of SOD and CAT in *T. thermophila* (Fig. 3). The SOD and CAT enhance the basal antioxidant capacity to overcome oxidative stress caused by Cd [43]. GSH acts as substrate for GPX to catalyze H_2_O_2_ [39]. The GSH content was notable upregulated under Cd stress, and with the addition of NaHS, the GSH content further increased (Fig. 3). The improvement of GSH levels and increase of SOD and CAT activities under NaHS treatment promoted detoxification of ROS, and protected the cells from oxidative damage. In addition, the accumulation of MDA content significantly reduced by the exogenous NaHS treatment. The exogenous NaHS promotes endogenous H_2_S formation and indirectly balances the O_2_^−^ and H_2_O_2_ levels and maintains redox balance in *T. thermophila*.

Cd stress leads to the significant molecular and physiological changes in different organisms. The whole transcriptional reprogramming is regarded as a vital molecular response to Cd stress. Enzymes responsible for detoxification and antioxidant process can alleviate oxidative stress. GPX protects organisms from oxidative damage by catalyzing the reaction between ROS and GSH [44]. Under arsenate stress, glutathione peroxidase family protein was over-expressed in *T. pyriformis*, which is involved in protecting the cells from oxidative damage [45]. Oxidoreductases participate in the oxidation-reduction process, and Cd exposure led to the remarkable variations of these genes in crayfish hepatopancreas [46]. Under Cd stress, 27 oxidoreductases and 9 glutathione peroxidase family proteins were upregulated in the oxidation–reduction process enrichment term in *T. thermophila* through GO analysis. Among them, the expression levels of 25 oxidoreductases and the all 9 glutathione peroxidase family proteins returned to normal upon the NaHS addition. The redox system was disrupted after Cd exposure and recovered through NaHS supplement in *T. thermophila.*

MTs are sulfhydryl-rich proteins, which have been known as multifunctional proteins and as pivotal members of the cellular integrated stress response system. *T. thermophila* has three CdMTs (*MTT1*, *MTT3*, and *MTT5*) and two CuMTs (*MTT2* and *MTT4*). *MTT5* is the strongest induced, while *MTT3* is the least induced by Cd stress at 1 h or 24 h [40, 47]. However, the ranking of relative expression values can change relying on the duration of heavy metal treatment [14]. We found that the ranking of induction fold change values was *MTT5*≈*MTT3*>*MTT1* when *T. thermophila* was stressed by Cd stress at 6 h (Fig. 8, and Table S2). Comparisons of qRT-PCR values obtained by different researchers are not similar due to differences in the experimental conditions. MTs also display oxyradical scavenging capacity and specifically neutralize hydroxyl radicals [48]. Interestingly, the upregulated expression of *MTT1* under Cd stress further increased with NaHS treatment. On the contrary, the upregulated expression of *MTT3* under Cd stress decreased with NaHS treatment. Meanwhile, the upregulated expression of *MTT5* under Cd stress has no significant change with NaHS treatment. These data further indicated that different MT genes have conserved and various function and regulated by different molecular mechanism.

To protect the cell from oxidative stress, heat shock proteins (HSPs) are greatly induced. In response to Cd exposure for 24 h in *Tetmemena*, there was around 46-fold increase in the hsp70 transcriptional expression as compared to control [21]. In the present study, three HSPs (TTHERM_000351139, TTHERM_00351140, TTHERM_00171850) were found to activate by Cd and the expression level returns to normal by NaHS addition (Fig. 8, and Table S2). It seems that H_2_S involved in the detoxification of Cd through the antioxidative stress signal pathway.

### H_2_S alleviates Cd stress by regulating cytochrome P450, GST and ABC transporters

The metabolism of xenobiotics comprises of three phases, including modification (phase I), conjugation (phase II), and excretion (phase III). Toxicity responsive genes of *T. thermophila* has been partially revealed, including cytochrome P450 gene family in phase I, GST gene family in phase II, and the ABC transporter superfamily in phase III [19]. Cytochrome P450 is in charge of the metabolism of most xenobiotics and efficiently eliminates foreign chemicals [36]. Cytochrome P450 was induced at 6 h of Cd treatment, then decreased to control levels after 18–24 h, and expressed at low level after 48 h of Cd exposure in *Physcia adscendens* [49]. We found that Cd altered the expression of 15 cytochrome P450 genes, and NaHS reversed the expression of most of these genes in *T. thermophila*.

P450 enzymes and GSTs are a well-conserved mechanism of the Cd response in a wide range of organisms [35]. GSTs are a large protein family with a well-established role in the conjugation of xenobiotics with GSH [50]. GSH forms complexes with heavy metals and serves as the first line of defense against heavy metals stress [51]. GST plays important roles through GSH metabolism in the protection against oxidative damage resulting from the high levels of ROS under Cd stress in *Chironomus riparius* [52]. In *Euplotes crassus*, copper and zinc significantly increased the expression of GST [53]. In animals, Cd is a redox-inactive metal that binds to the thiol group of GSH [54]. The expression level of 31 GSTs was upregulated under Cd stress. Among them, the expression level of 29 GSTs was downregulated with NaHS addition in *T. thermophila.*

GSH marked xenobiotic molecules are transported via phase III proteins such as ABC transporters. The ABC transporters comprise large protein families that mediate various physiological processes [55]. In fission yeast, Cd forms complexes with either GSH or PCs and subsequently transports into vacuoles via the ABC transporters. The ABC transporters in plants transport GS_2_–Cd or PC–Cd complexes into subcellular compartments or out of cells [56]. *MRP1* is a well-known ABCC family gene in human, and also regarded as GS-X pumps [57]. Moreover, *MRP5*, another ABC transporter C family gene, has an important role in the detoxification of Cd [58]. MRP proteins are potential mediators of heavy metal resistance in zebrafish cells [59]. *ABCC52* is a MRP-like gene in *T. thermophila* and induced by Cd and recovered through NaHS treatment. In *T. thermophila*, the membrane pump protein encoded by ABCT gene abcb15 enhance the tolerance of DDT and protect cells from this exogenous toxin by efficiently pumping the toxin to the extracellular space [60]. The *ABCB15* of *T. thermophila* shares a 32.3% amino acid identity with *ABCB1* in human, which has been shown to confer resistance to or transport a wide variety of toxic compounds [18]. The induction of *ABCB1* was detected in *Euglena* exposed to Cd [61]. Exposure to Cd in the gills and hepatopancreas of oyster showed activation of *ABCB1* protein but no significant changes to its mRNA, suggesting post-transcriptional regulation of *ABCB1* [62]. We do not detect the change of *ABCB15* at transcriptional level under Cd stress in *T. thermophila*, and the post-transcriptional regulation needs further study. As a whole, a total of 67 DEGs were enriched in the ABC transporter pathway under Cd stress, and the ABC transporter pathway was significantly altered with NaHS addition. So, the decrease of Cd accumulation and toxicity under NaHS treatment partially attribute to regulation of CdS nanoparticles transport by ABC transporter pathway in *T. thermophila* cells.

## Conclusion

H_2_S physiologically alleviated growth inhibition under Cd stress and accumulation of Cd in *T. thermophila*. H_2_S promoted formation of CdS nanoparticles and decreased Cd bioavailability and toxicity, which mitigated lipid peroxidation and improved GSH levels as well as SOD and CAT activities for detoxification of ROS. H_2_S affected various metabolic processes and molecular functions under Cd stress, including glutathione metabolism, metabolism of xenobiotics by cytochrome P450, GPXs, Cytochrome P450s, GSTs, and ABC transporters. Taken together, H_2_S protects *T. thermophila* from Cd stress by inhibiting Cd absorbtion, promoting CdS nanoparticles formation, increasing oxidation resistance, and regulation of transport.

## Methods

### Strains and culture conditions

*T. thermophila* B2086 was obtained from the *Tetrahymena* Stock Center (http://tetrahymena.vet.cornell.edu/, Cornell University, Ithaca). The cells were cultured in a 1×SPP medium (pH 7.4) containing 1% proteose peptone, 0.1% glucose, 0.2% yeast extract, and 33.3 μM EDTA-FeNa_2_ at 30 °C with continuous shaking [63]. For physiological and biochemical analyses, cells were transferred to 10 mL of SPP medium with the same initial concentration at 0.7× 10^5^ mL^− 1^.

### Cellular proliferation analysis

Various Cd concentrations ranging from 0.5 μM to 256 μM were used to determine the IC50 of Cd for *T. thermophila* cells for 6 h treatment. The S-curve was plotted with GraphPad Prism 5 in terms of inhibition rates and Cd concentrations. In a pilot study, 6 levels of NaHS (donor of H_2_S) concentrations (0, 50, 70, 100, 200, 400 μM) were explored for treating *T. thermophila* cells. Based on the results of the pilot study, 70 μM NaHS was identified as the optimum positive effect H_2_S concentration, which was subsequently used as the processing condition in the subsequent experiment (Fig. 1b). To analyze the effects on cell proliferation of H_2_S under Cd stress, cells were uniformly grown in the SPP medium with 30 μM Cd (IC50) in the presence or absence of 70 μM NaHS (optimum positive effect concentration), and their starting concentration was 0.7× 10^5^ mL^− 1^. Each treatment was replicated 3 times, and the sample was taken every 4 h. The number of cells was determined using a blood cell counting chamber. The formation of CdS by NaHS and CdCl_2_ in vitro was negligible at present low Cd and H_2_S concentrations [29].

### Measurement of H_2_S and cysteine contents

The cells were collected by centrifugation, and lysates were prepared by sonication. The crude extracts were centrifuged at 12,000 g for 10 min at 4 °C, and H_2_S quantification was performed according the instruction in the H_2_S assay kit (Nanjing Jiancheng Bioengineering Institute, China). Cysteine content was determined as described previously [64].

### Measurement of Cd concentration

*T. thermophila* cells were cultured with 30 μM Cd alone or with the addition of 70 μM NaHS treatments for 24 h. After centrifugation, the cells that accumulated Cd were removed from the medium. The Cd in the medium was measured using inductively coupled plasma atomic emission spectroscopy (ICP-OES, iCAP 7000 Series, Thermo Scientific). The results represented the average of three independent experiments.

### Assay of MDA, GSH, SOD, and CAT

The cells (3× 10^6^) were exposed to various Cd concentrations for 6 h or 70 μM NaHS before they were collected and ultrasonically lysed. The lysate was centrifuged at 12,000 g for 20 min at 4 °C. The protein quantitation was evaluated through the bicinchoninic acid (BCA) assay. The amount of lipid peroxidation product MDA was determined by the thiobarbituric acid (TBA) method according to the reaction of MDA with TBA [65]. Each experiment was done in triplicate.

The cells in the logarithmic phase were divided into four groups with different treatments: (1) CK, without chemical treatments (the standard SPP solution); (2) S, treatment with NaHS (70 μM); (3) S+HT, treatment with NaHS (70 μM) and HT (140 μM); (4) C, treatment with Cd (30 μM); and (5) C+S, treatment with Cd (30 μM) and NaHS (70 μM); (6) C+S+HT, treatment with Cd (30 μM), NaHS (70 μM), and HT (140 μM). After treatment for 6 h, the cells were centrifuged and ultrasonically lysed. The antioxidant contents of GSH were determined by reacting with 5, 5′-dithiobis-(2-nitrobenzoic acid) (DTNB), and the yellow products were evaluated by colorimetric method at 405 nm according to the instruction in the GSH assay kit (Nanjing Jiancheng Bioengineering Institute, china). A microtiter plate assay was used to determine the SOD activities utilizing a water-soluble tetrazolium salt (WST-1), which was used as a detector of superoxide radical generated by xanthine oxidase and hypoxanthine [66]. The SOD activity was measured with the standard SOD assay kit (Nanjing Jiancheng Bioengineering Institute, China). One unit of enzymatic activity caused 50% inhibition of WST-1 formazan. CAT activities were determined by Goth’s colorimetric method. The supernatant was incubated with H_2_O_2_ substrate, and the enzymatic reaction stopped by the addition of ammonium molybdate. The intensity of the yellow complex was formed by molybdate and measured at 405 nm [67]. One unit of activity indicated 1 μmol of decomposition of H_2_O_2_ per second. Each experiment was repeated thrice.

### Observation of intracellular CdS nanoparticles

Cells were visualized with light microscope Olympus BX51 equipped with DP70 camera to monitor the cells for any changes in the appearance after treatment with Cd or Cd and NaHS.

### Spectral analyses

UV–visible absorbance spectra were recorded from 200 nm to 700 nm in the presence or absence of Cd or NaHS in solution using a PerkinElmer Lambda 35 spectrometer. Each spectrum represents the average of three individual scans.

### Extraction of total RNA

A total of 3× 10^6^ cells were harvested from the CK, S, C, CS samples with 3 replications, immediately frozen in liquid nitrogen, and stored at − 80 °C. Total RNA was manually extracted using a TRIzol regent (Invitrogen, USA) in accordance with the manufacturer’s instructions. RNA contamination and degradation were monitored on 1% agarose gels. RNA purity was checked with a NanoPhotometer® spectrophotometer (IMPLEN, CA, USA). RNA concentration was measured with a Qubit® RNA assay kit in a Qubit®2.0 fluorometer (Life Technologies, CA, USA). RNA integrity was evaluated using an RNA Nano 6000 assay kit of an Agilent Bioanalyzer 2100 system (Agilent Technologies, CA, USA).

### Library construction, illumina sequencing, and transcriptome assembly

A total amount of 3 μg RNA was used as input material for the RNA sample preparations. Sequencing libraries were generated using a NEBNext®Ultra™ RNA library prep kit for Illumina® (NEB, USA) in accordance with the manufacturer’s instructions, and index codes were added to attribute the sequences to each sample. mRNA was purified from the total RNA using poly-T oligo-attached magnetic beads. Fragmentation was conducted using divalent cations at an increased temperature in a NEBNext first-strand synthesis reaction buffer (5×). First-strand cDNA was synthesized using a random hexamer primer and M-MuLV reverse transcriptase (RNase H). Second-strand cDNA was subsequently synthesized using DNA polymerase I and RNase H. The remaining overhangs were converted into blunt ends via exonuclease/polymerase activities. After the 3′ ends of the DNA fragments were adenylated, a NEBNext adaptor with a hairpin loop structure was ligated to prepare for hybridization. The library fragments were purified with an AMPure XP system (Beckman Coulter, Beverly, USA) to select the cDNA fragments with preferentially 150–200 bp in length. Then, 3 μL of USER Enzyme (NEB, USA) was used with a size-selected, adaptor-ligated cDNA initially at 37 °C for 15 min and then at 95 °C for 5 min. Then, PCR was performed with Phusion high-fidelity DNA polymerase, universal PCR primers, and index (X) primer. PCR products were purified (AMPure XP system), and library quality was assessed using the Agilent Bioanalyzer 2100 system. The index-coded samples were clustered on a cBot cluster generation system using a TruSeq PE cluster kit v3-cBot-HS (Illumina) in accordance with the manufacturer’s instructions. After generating the cluster, the prepared libraries were sequenced on an Illumina Hiseq 2000 platformto generate paired-end reads. Transcriptome assembly was accomplished using Trinity [68] with min_kmer_cov set to 2 by default. All of the other parameters were set to default.

### Gene expression level quantification and differential expression analysis

Gene expression levels were estimated by RSEM [69] for each sample. Clean data were mapped on the assembled transcriptome, and the read count for each gene was obtained from the mapping results. Two conditions/groups were subjected to differential expression analysis using the DESeq R package (1.10.1). The resulting *P* values were adjusted using Benjamini and Hochberg’s approach for controlling the FDR. Genes with an adjusted *P* < 0.05 and |log2 (fold-change)| > 1 found by DESeq were assigned as differentially expressed. The GO enrichment analysis of DEGs was implemented by the topGO R packages in a Kolmogorov–Smirnov (KS) test. The KEGG [70] was used to understand the functions of cells at the molecular-level (http://www.genome.jp/kegg/). KOBAS was used to evaluate the statistical enrichment of DEGs in KEGG pathways [71].

### qRT-PCR validation

The total RNAs of the four different groups were extracted from the frozen cell samples with RNAiso Plus (TaKaRa, Japan). Total RNA (2 μg) was reverse-transcribed into first-strand cDNA using PrimeScript™ RT reagent kit with gDNA Eraser (Takara, Japan). qRT-PCR was conducted using an SYBR Green II (SYBR® Premix Ex Taq™ Kit, Takara) in a CFX96TM real-time system (BIO-RAD, USA) through the following steps: heat for 30 s at 95 °C, followed by 40 cycles of 5 s at 95 °C and extension for 30 s at 60 °C. A melting curve analysis was performed after every PCR reaction to confirm the accuracy of each amplified product. The 17S rRNA gene was used as an internal control. All primers used for qRT-PCR are listed in Table S4. Amplification efficiency (E) was measured by using 4-fold serial dilutions of a positive control PCR template. The efficiency requirement was met for all the tested genes. Results were finally processed by the standard-curve method [72]. Their amplification efficiencies were greater than 90% and the correlation coefficients (R^2^) were all 99% (Table S5). The relative expression levels of genes were calculated using the 2^−ΔΔCT^ method [73]. Three independent replicates were performed for each treatment. The correlation analysis of qRT-PCR and RNA-seq were based on Pearson’s correlation coefficient.

### Statistical analysis

Each treatment was conducted in three replicates. Data were subjected to an analysis of variance (ANOVA). Duncan’s multiple range test was used to compare significant differences at *P* < 0.05 among various treatments. Different letters indicated significantly different results.

## Supplementary Information

**Additional file 1: Figure S1.** Changes of MDA content in various Cd concentrations. Data are means ± SE of three biological repeats, error bars indicate error standard. Means denoted by the same letter were not significantly different at *P* > 0.05, and different letters indicate statistically significantly differences (*P* < 0.05) by Duncan Multiple Range Test (DMRT).

**Additional file 2: Figure S2.** Absorbance spectra were taken from 200 nm to 700 nm in the presence or absence of Cd or NaHS in solution. Each spectrum represents the average of three individual scans.

**Additional file 3: Table S1.** Summary for the mapped information.

**Additional file 4: Figure S3.** Correlation heatmap of samples. The gradient color barcode on the left presents the minimum value in pink and the maximum in blue. If one sample is highly similar to another one, the correlation between them is close to 1.

**Additional file 5: Table S2.** DEGs of log2FC > 8 in CK vs. C based on RNA-seq analysis.

**Additional file 6: Table S3.** DEGs of log2FC < − 8 in C vs. CS based on RNA-seq analysis.

**Additional file 7: Figure S4.** GO functional analysis of DEGs. GO functional categories of DEGs in CK vs. C and C vs. CS. The ordinate coordinates represent the gene numbers and percentages in the background of all genes or DEGs. Three different classifications of the GO annotations under three basic categories are included (from left to right: biological processes, cellular component, and molecular function).

**Additional file 8: Figure S5.** Classification of DEGs based on KEGG analysis with respect to CK vs. C (**a**) and C vs. CS (**b**). The vertical axis represents the name of the pathway, and the horizontal axis denotes the number of genes.

**Additional file 9: Figure S6.** Heat-map of metabolism of xenobiotics by cytochrome P450; the colors from green to red represent the gene express values (FPKM) from low to high. The three columns represent the three experimental groups. CK represents the control group with no Cd or NaHS treatment. C represents the group treated by Cd. CS represents the group with NaHS treatment under Cd stress. The genes included enriched DEGs in CK vs. C and/or C vs. CS based on KEGG analysis. Genes IDs are on the right.

**Additional file 10: Figure S7.** Heat-map of GSH metabolism; the colors from green to red represent the gene express values (FPKM) from low to high. The three columns represent the three experimental groups. CK represents the control group with no Cd or NaHStreatment. C represents the group treated by Cd. CS represents the group with NaHS treatment under Cd stress. The genes included enriched DEGs in CK vs. C and/or C vs. CS based on KEGG analysis. Genes IDs are on the right.

**Additional file 11: Figure S8.** Heat-map of ABC transporters; the colors from green to red represent the gene express values (FPKM) from low to high. The three columns represent the three experimental groups. CK represents the control group with no Cd or NaHS treatment. C represents the group treated by Cd. CS represents the group with NaHS treatment under Cd stress. The genes included enriched DEGs in CK vs. C and/or C vs. CS based on KEGG analysis. Genes IDs are on the right.

**Additional file 12: Figure S9.** Cell proliferation under 70 μM NaHS, 1.2 mM Cu, and 1.2 mM Cu + 70 μM NaHS treatments.

**Additional file 13: Table S4.** Primers used in this study.

**Additional file 14: Table S5.** qRT-PCR standard-curve parameters.

## Data Availability

All data supporting the conclusions of this article are provided within the article and its supplementary files. All RNA-Seq data are available in the NCBI Sequence Read Archive database under BioProject accession number PRJNA684669 (http://www.ncbi.nlm.nih.gov/bioproject/684669).

## Abbreviations

Cd: Cadmium
H_2_S: Hydrogen sulfide
NaHS: Sodium hydrosulfide
*T. thermophila*: *Tetrahymena thermophila*
ROS: Reactive oxygen species
O^•^_2_^−^: Superoxide anion
OH^•^: Hydroxyl radical
H_2_O_2_: Hydrogen peroxide
SOD: Superoxide dismutase
CAT: Catalase
GSH: Glutathione
GPX: Glutathione peroxidase
GST: Glutathione S-transferase
CBS: Cystathionine β-synthase
CGL: Cystathionine γ-lyase
CS: Cysteine synthase
MDA: Malondialdehyde
MTs: Metallothioneins
PCs: Phytochelatins
IC50: Half maximal inhibitory concentration
CK: Control
S: Control with H_2_S treatment
C: Control under Cd stress
CS: Control with H_2_S treatment under Cd stress
DEGs: Differentially expression genes
FC: Fold Change
FDR: False discovery rate
FPKM: Fragments per kilo base million
KS: Kolmogorov–Smirnov
GO: Gene ontology
KEGG: Kyoto Encyclopedia of Genes and Genomes
qRT-PCR: Quantitative real-time polymerase chain reaction
E: Amplification efficiency
R^2^: Correlation coefficient
