# Supplementary figures and images for: Comparative transcriptome analysis uncovers roles of hydrogen sulfide for alleviating cadmium toxicity in Tetrahymena thermophila
Source: BMC Genomics. 2021 Jan 6;22:21. doi: 10.1186/s12864-020-07337-9 (PMC7788932; doi:10.1186/s12864-020-07337-9)

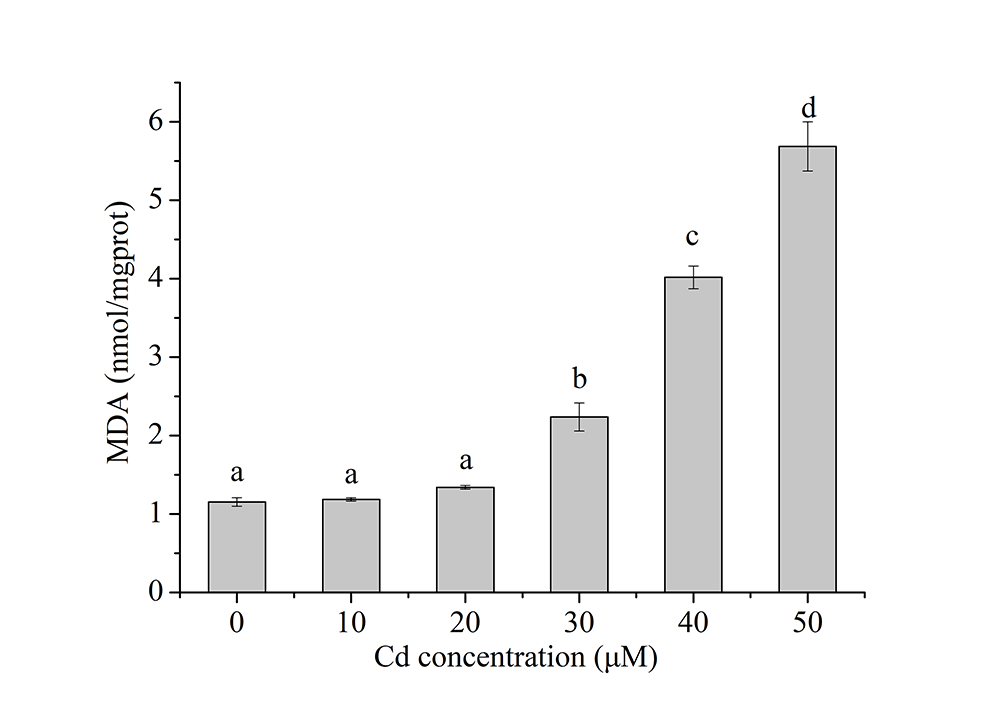

Supplement: Supplementary file 1 — Additional file 1: Figure S1. Changes of MDA content in various Cd concentrations. Data are means ± SE of three biological repeats, error bars indicate error standard. Means denoted by the same letter were not significantly different at P > 0.05, and different letters indicate statistically significantly differences (P < 0.05) by Duncan Multiple Range Test (DMRT). [file 12864_2020_7337_MOESM1_ESM.tif]

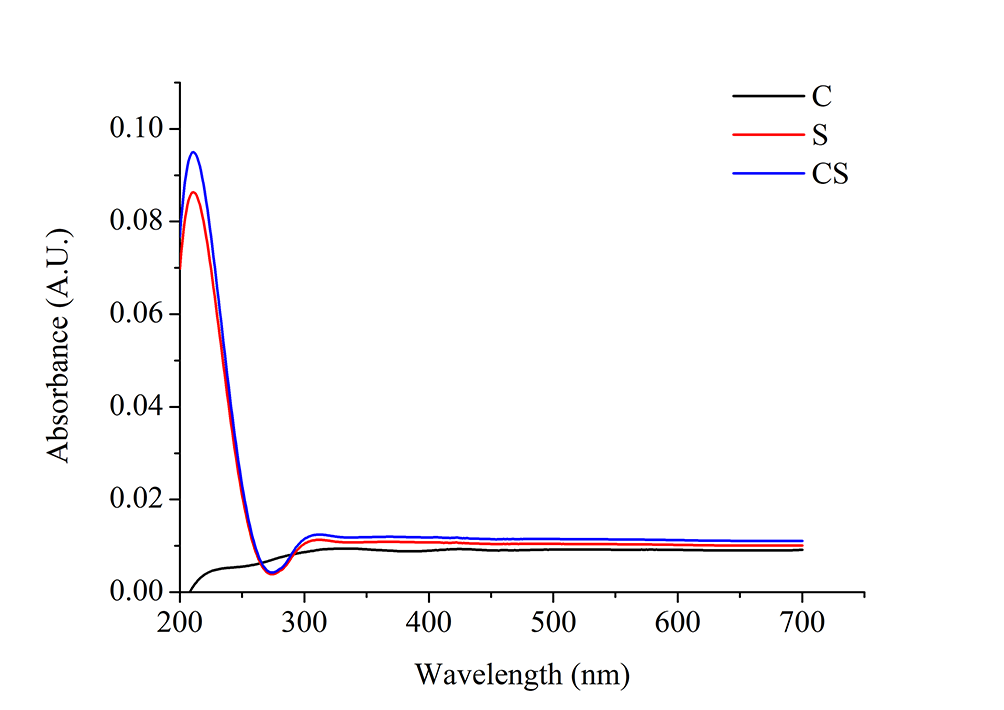

Supplement: Supplementary file 2 — Additional file 2: Figure S2. Absorbance spectra were taken from 200 nm to 700 nm in the presence or absence of Cd or NaHS in solution. Each spectrum represents the average of three individual scans. [file 12864_2020_7337_MOESM2_ESM.tif]

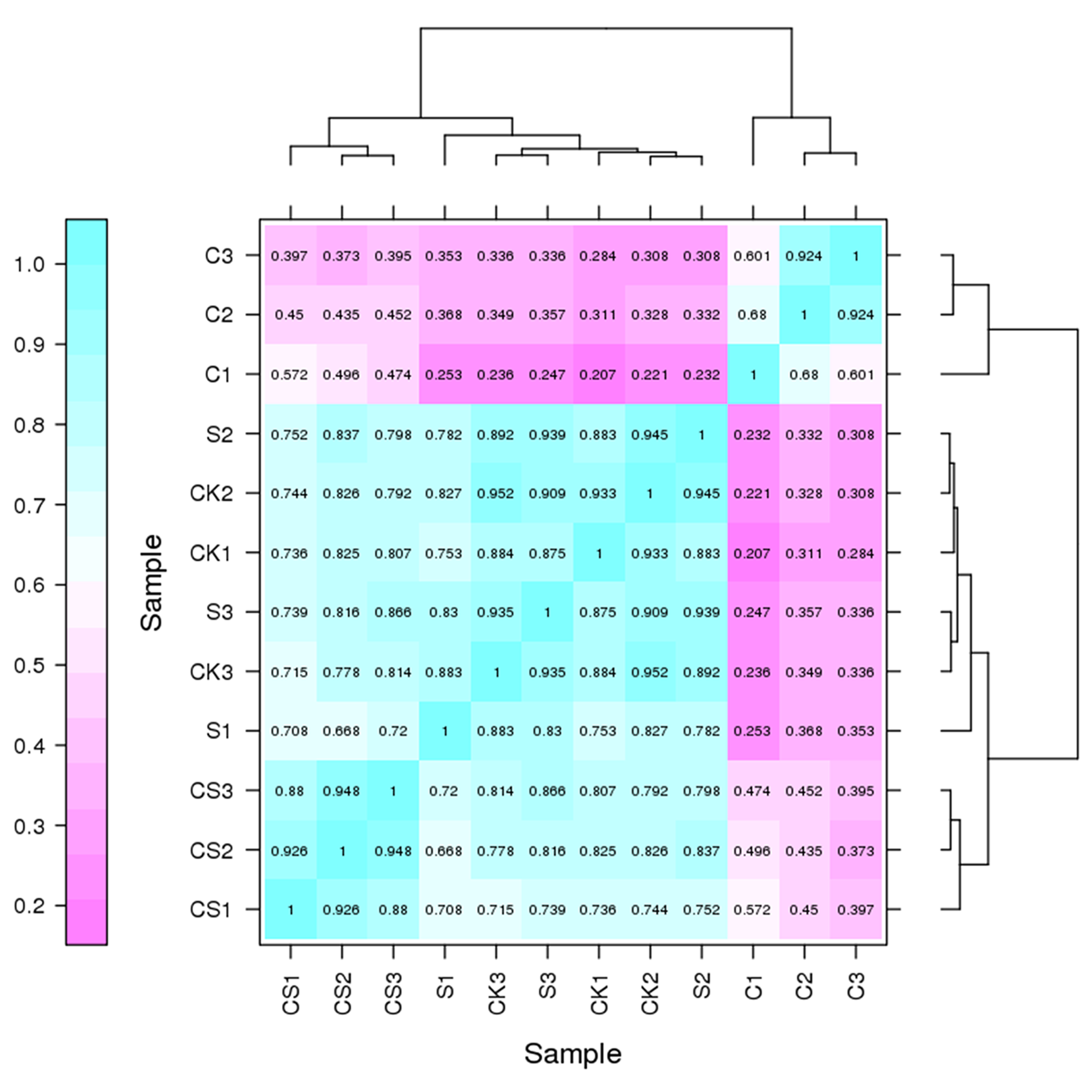

Supplement: Supplementary file 4 — Additional file 4: Figure S3. Correlation heatmap of samples. The gradient color barcode on the left presents the minimum value in pink and the maximum in blue. If one sample is highly similar to another one, the correlation between them is close to 1. [file 12864_2020_7337_MOESM4_ESM.tif]

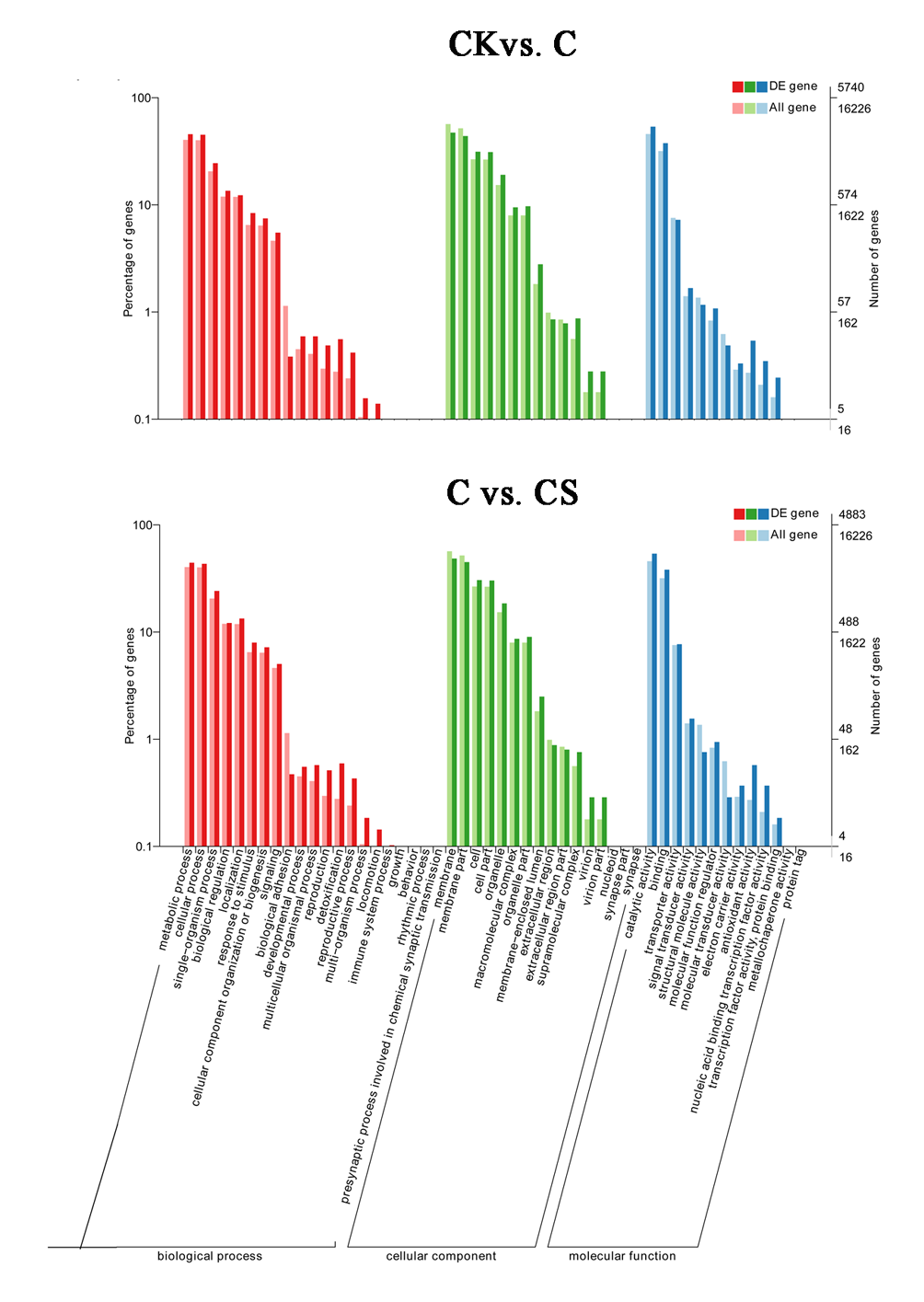

Supplement: Supplementary file 7 — Additional file 7: Figure S4. GO functional analysis of DEGs. GO functional categories of DEGs in CK vs. C and C vs. CS. The ordinate coordinates represent the gene numbers and percentages in the background of all genes or DEGs. Three different classifications of the GO annotations under three basic categories are included (from left to right: biological processes, cellular component, and molecular function). [file 12864_2020_7337_MOESM7_ESM.tif]

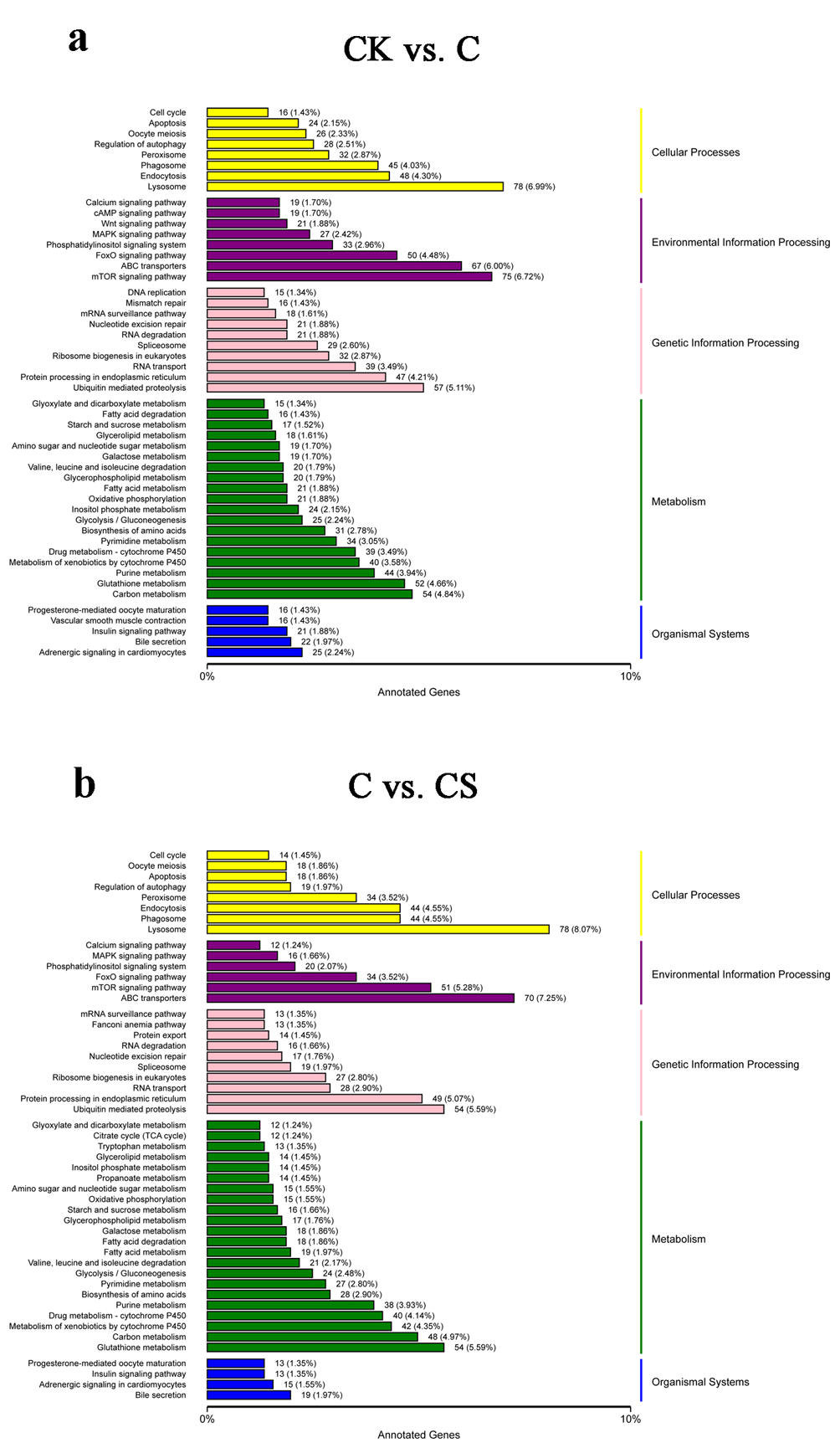

Supplement: Supplementary file 8 — Additional file 8: Figure S5. Classification of DEGs based on KEGG analysis with respect to CK vs. C (a) and C vs. CS (b). The vertical axis represents the name of the pathway, and the horizontal axis denotes the number of genes. [file 12864_2020_7337_MOESM8_ESM.tif]

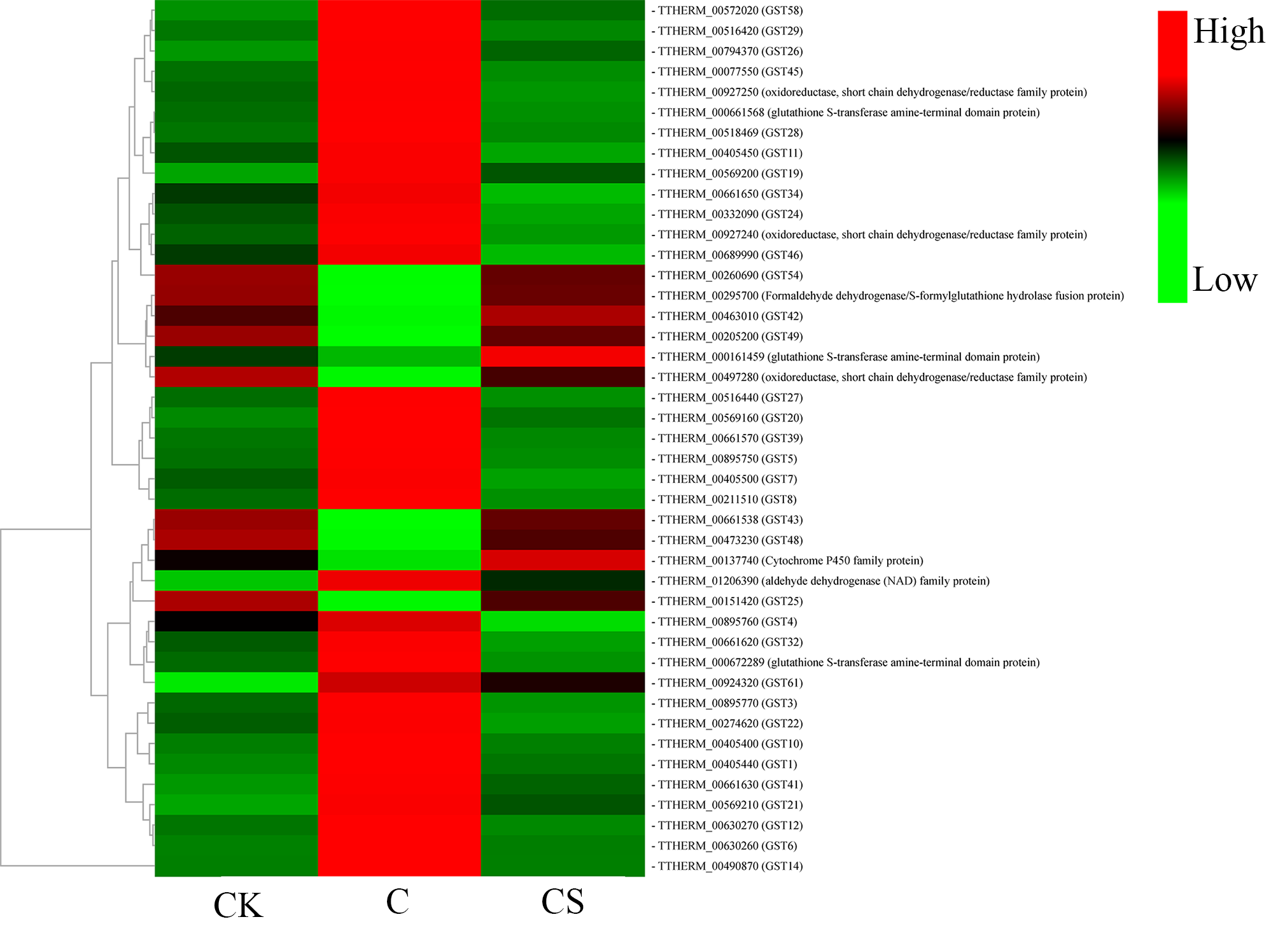

Supplement: Supplementary file 9 — Additional file 9: Figure S6. Heat-map of metabolism of xenobiotics by cytochrome P450; the colors from green to red represent the gene express values (FPKM) from low to high. The three columns represent the three experimental groups. CK represents the control group with no Cd or NaHS treatment. C represents the group treated by Cd. CS represents the group with NaHS treatment under Cd stress. The genes included enriched DEGs in CK vs. C and/or C vs. CS based on KEGG analysis. Genes IDs are on the right. [file 12864_2020_7337_MOESM9_ESM.tif]

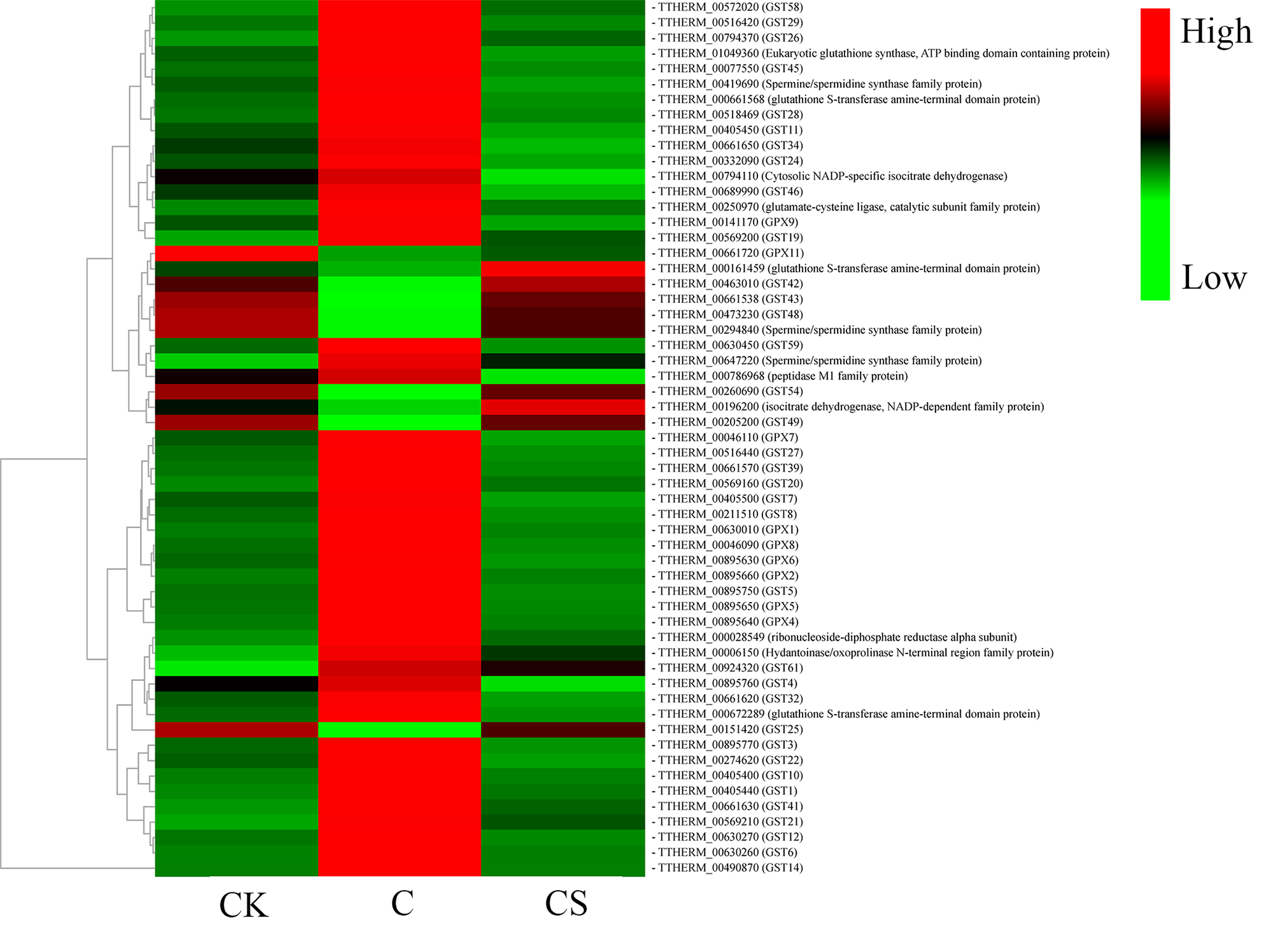

Supplement: Supplementary file 10 — Additional file 10: Figure S7. Heat-map of GSH metabolism; the colors from green to red represent the gene express values (FPKM) from low to high. The three columns represent the three experimental groups. CK represents the control group with no Cd or NaHStreatment. C represents the group treated by Cd. CS represents the group with NaHS treatment under Cd stress. The genes included enriched DEGs in CK vs. C and/or C vs. CS based on KEGG analysis. Genes IDs are on the right. [file 12864_2020_7337_MOESM10_ESM.tif]

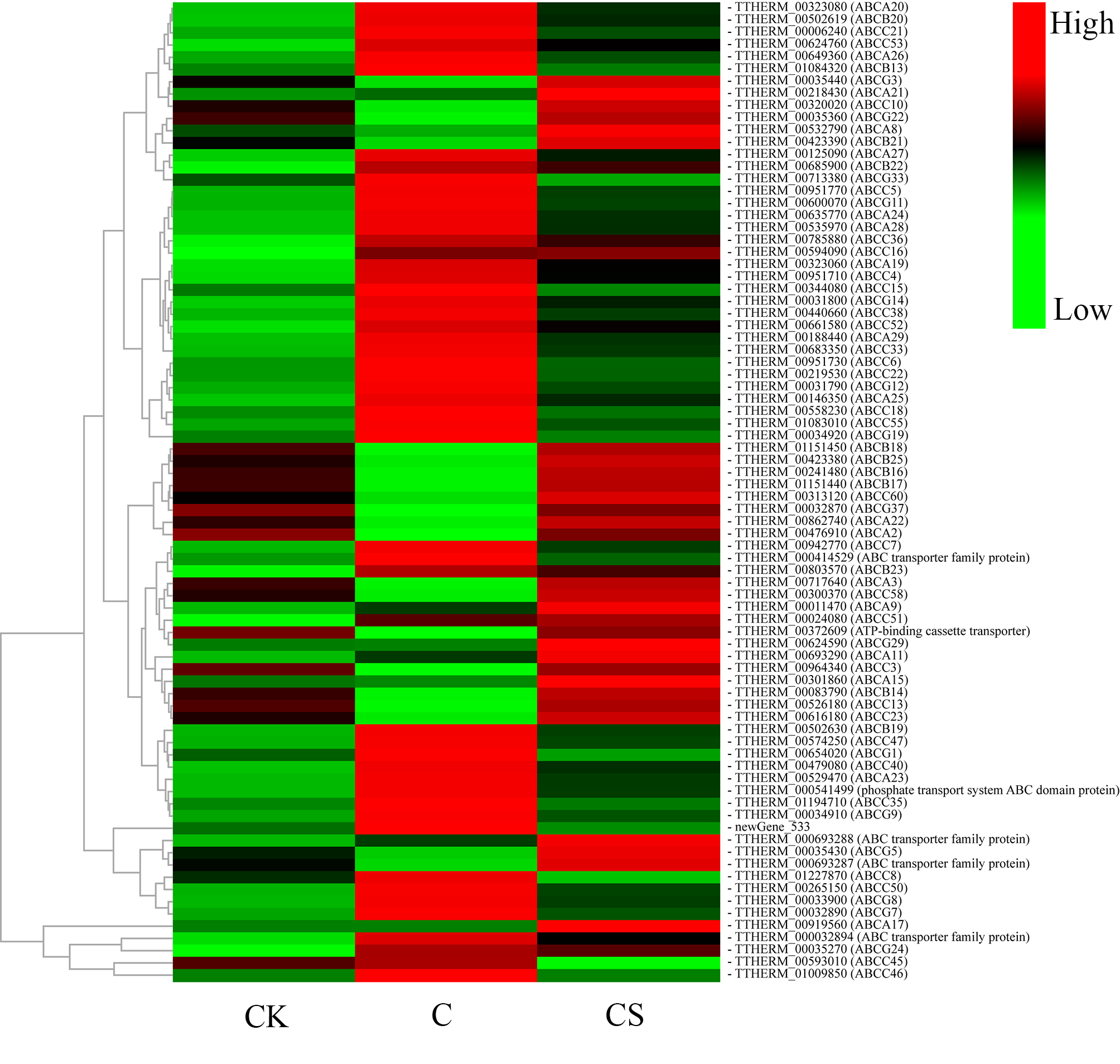

Supplement: Supplementary file 11 — Additional file 11: Figure S8. Heat-map of ABC transporters; the colors from green to red represent the gene express values (FPKM) from low to high. The three columns represent the three experimental groups. CK represents the control group with no Cd or NaHS treatment. C represents the group treated by Cd. CS represents the group with NaHS treatment under Cd stress. The genes included enriched DEGs in CK vs. C and/or C vs. CS based on KEGG analysis. Genes IDs are on the right. [file 12864_2020_7337_MOESM11_ESM.tif]

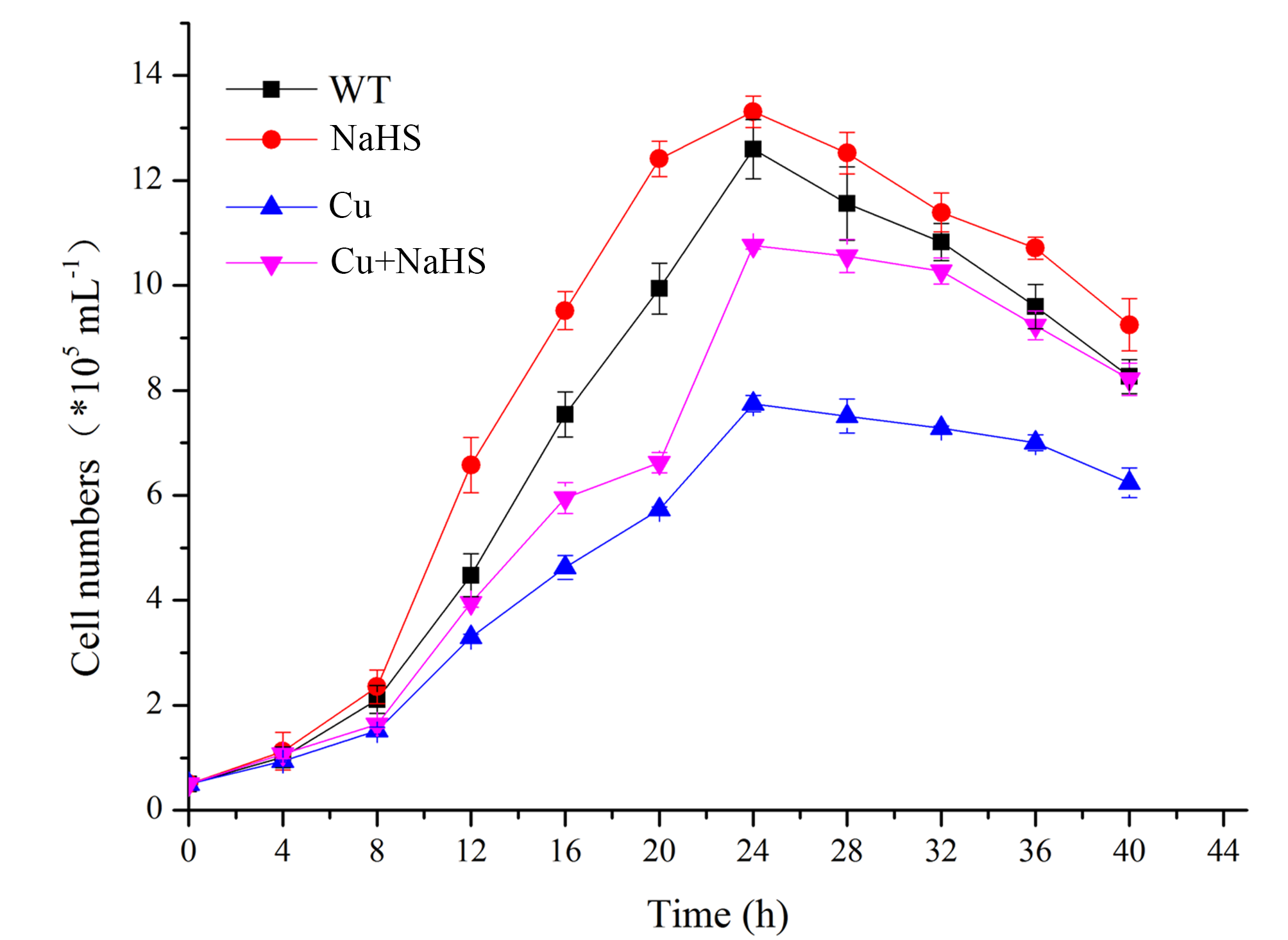

Supplement: Supplementary file 12 — Additional file 12: Figure S9. Cell proliferation under 70 μM NaHS, 1.2 mM Cu, and 1.2 mM Cu + 70 μM NaHS treatments. [file 12864_2020_7337_MOESM12_ESM.tif]
